# Systemic hypertension is not protective against chronic intraocular pressure elevation in a rodent model

**DOI:** 10.1038/s41598-018-25264-4

**Published:** 2018-05-08

**Authors:** Anna K. van Koeverden, Zheng He, Christine T. O. Nguyen, Algis J. Vingrys, Bang V. Bui

**Affiliations:** Department of Optometry and Vision Sciences, the University of Melbourne, Parkville, 3010 Victoria Australia

## Abstract

High intraocular pressure is the most well documented glaucoma risk factor; however many patients develop and/or show progression of glaucoma in its absence. It is now thought that in some instances, ocular perfusion pressure (blood pressure – intraocular pressure) may be as important as intraocular pressure alone. Thus, systemic hypertension would be protective against glaucoma. Epidemiological studies, however, are inconclusive. One theory of why hypertension may not protect against elevated intraocular pressure in spite of increasing ocular perfusion pressure is that with time, morphological changes to the vasculature and autoregulatory failure outweigh the benefits of improved perfusion pressure, ultimately leading to poor retinal and optic nerve head blood supply. In this study we showed the presence of increased wall:lumen ratio and wall area of the ophthalmic artery in rats with chronic hypertension in addition to failure of retinal autoregulation in response to acute modification of ocular perfusion pressure. Subsequently we found that in spite of dramatically increasing ocular perfusion pressure, chronic systemic hypertension failed to protect retinal structure and function from a rodent model of glaucoma.

## Introduction

Glaucoma is the second leading cause of blindness worldwide^1^, and the most well established glaucoma risk factor is raised intraocular pressure (IOP)^2,3^. In many cases however, glaucoma can develop and/or progress in its absence^4,5^. Thus, investigators have sought to understand the role of systemic risk factors in glaucoma. Emerging from these studies is the consistent finding that vascular risk factors can impact upon optic nerve head (ONH) and retinal blood supply and may be critical in glaucoma pathophysiology^6–11^. Systemic hypertension may be a critical factor in this vascular hypothesis of glaucoma, however its role is not without controversy^12^. Ocular perfusion pressure (OPP) is the difference between mean arterial pressure (MAP) in the ophthalmic artery and IOP. As the retina does not store glucose, adequate tissue perfusion is critical to maintaining retinal function, and as the eye is subjected to constantly changing OPP, it relies on vascular autoregulation to buffer this variation^13–15^. Experimental and clinical studies suggest that it is not necessarily excessive IOP that leads to glaucoma, rather insufficient OPP, which may arise by virtue of either high IOP or low BP (blood pressure)^16–18^. As Khawaja *et al*.^19^ highlighted, the relationship between BP, IOP and OPP is complex, and it is somewhat difficult to isolate the contribution of BP or IOP as part of OPP towards glaucoma risk.

There is strong evidence that low BP increases glaucoma risk^20,21^. Paradoxically, a number of population based studies also find that systemic hypertension increases glaucoma risk^22–25^. The reason for this apparent contradiction may be that whilst low BP promotes hypoperfusion of the retina and optic nerve, chronic periods of hypertension may also promote poor blood supply due to the development of arteriosclerosis and autoregulatory failure. As OPP is not a static entity, adequate autoregulatory capacity, achieved by reflexive alterations in vascular resistance, is necessary to buffer changes in tissue perfusion pressure^13,26^.

The Baltimore Eye Survey found hypertension was protective against glaucoma in younger subjects and increased glaucoma risk in older subjects^22^. Additionally, both the Egna-Neumarkt and Los Angeles Latino Eye Studies found both hyper- and hypo-tension were associated with increased risk of glaucoma^23,25^. In particular low diastolic perfusion pressure and high systolic perfusion pressure carried an increased risk of glaucoma. These findings lend support to the hypothesis that whilst low OPP is undoubtedly detrimental, changes to the vasculature that occur as a result of long periods of hypertension may eventually undermine the benefit of increased OPP afforded by hypertension.

Experimental studies in rats find that high BP preserves retinal function during acute IOP challenge, however the degree of protection becomes blunted in the presence of a longer duration of hypertension (one hour vs four weeks)^16,27^. In animals with the same OPP, retinal susceptibility to injury was altered by the duration of systemic hypertension. Therefore it is not merely the perfusion pressure itself but the perfusion pressure status, encompassing perfusion pressure along with autoregulatory capacity and vascular morphology, which is important in maintaining adequate tissue perfusion. In our experiment, we sought to extend the duration of systemic hypertension further, to 12 weeks, in order to further explore the temporal aspects of the relationship between blood pressure and perfusion pressure status.

In addition to poor perfusion pressure, impaired autoregulation has been linked to glaucoma risk^18,28,29^. Cerebral autoregulation is known to be altered in chronic hypertension^30^. Additionally, a blunted response to a metabolic challenge of retinal autoregulation has been found in humans with chronic hypertension^31,32^. Changes to vascular morphology may underlie poor perfusion and autoregulatory deficits. In this study we sought to investigate the effect of chronic hypertension on retinal autoregulation in response to OPP modification. In these rats we also examined the morphology of the ophthalmic vasculature, as the mechanisms hypothesised to underlie a BP mediated altered susceptibility to high IOP. After investigating these effects, we aimed to test whether the benefits of improved OPP in hypertension would be outweighed by the altered perfusion pressure status when chronic systemic hypertension was overlaid with chronic ocular hypertension.

## Results

### Blood pressure profile

In the hypertensive group (continuous subcutaneous angiotensin II (ANG II) infusion), systolic blood pressure (SBP) increased steadily over the 12 weeks of infusion from a baseline of 123.0 ± 2.5 mmHg to 161.3 ± 4.6 mmHg at week 12 (see also Supplementary Figure S1). In control animals (continuous subcutaneous saline infusion), BP remained stable over the 12 weeks, relative to baseline (123.6 ± 2.8 mmHg). The average SBP from weeks 1–12 was 164.8 ± 4.6 mmHg and 122.6 ± 2.3 mmHg in hypertensive and normotensive animals, respectively.

### Effect of chronic hypertension on retinal autoregulatory capacity

**Figure 1:**
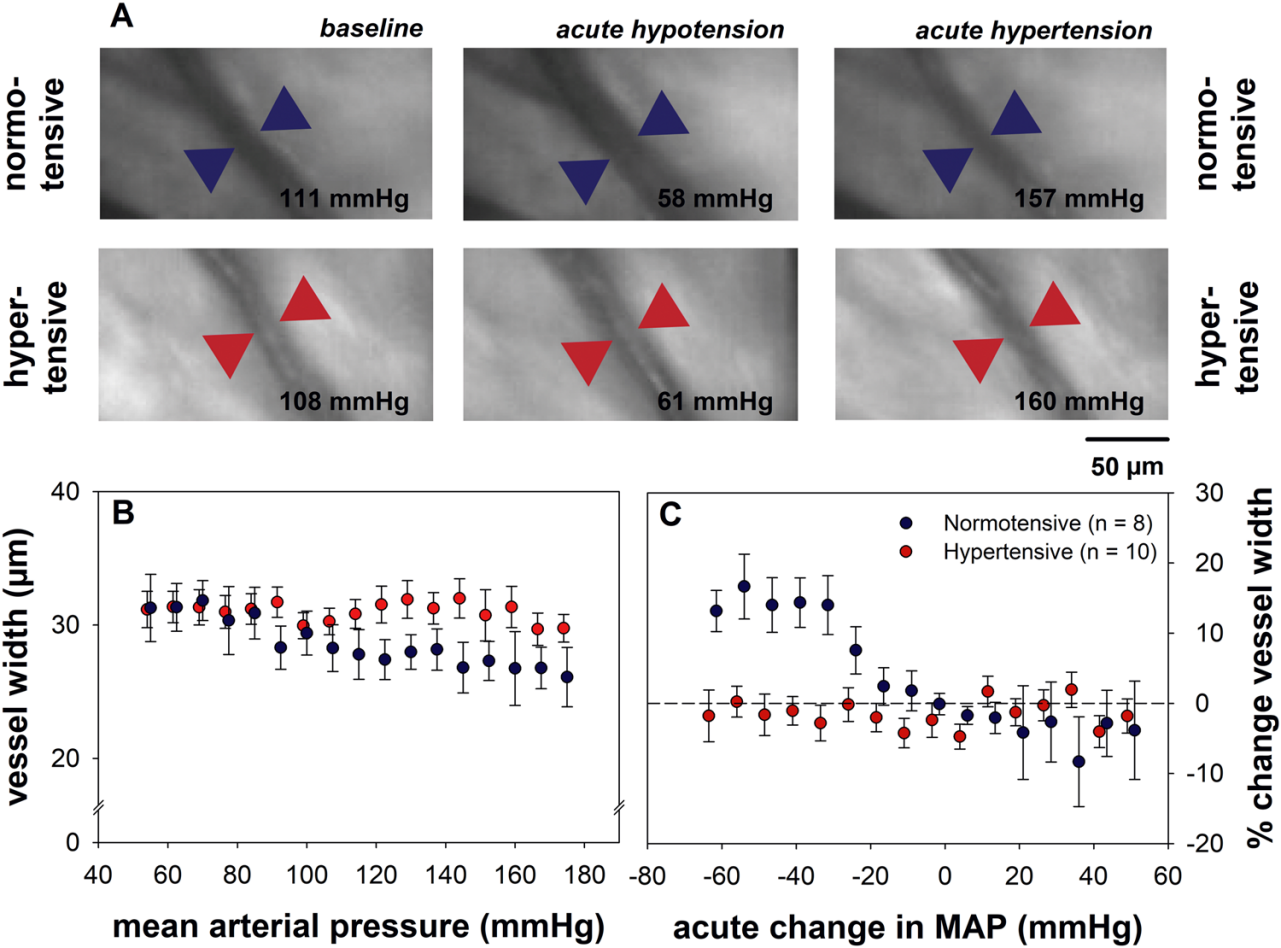
Effect of chronic hypertension on retinal blood vessel reactivity during acute BP manipulation. (**A**) Representative arterioles from a normotensive rat (blue arrowheads) and a hypertensive rat (red arrowheads) during BP manipulation procedure. The normotensive rat shows vasodilation relative to baseline during acute hypotension, and vasoconstriction during acute hypertension. The hypertensive rat show minimal change in vessel diameter. The mean arterial pressure at the time of blood vessel imaging is shown in the bottom right corner of each image. (**B**) Average arteriole width (mean ± SEM) in response to acute BP manipulation. (**C**) Change in arteriole width (mean ± SEM) from baseline as a function of change in MAP from baseline. The interaction between BP group and vasodilation was significant at changes in MAP from baseline between −70.0 and −32.5 mmHg.

After 12 weeks of systemic hypertension, animals in Group-1 underwent acute BP manipulation during which changes in retinal blood vessel diameter were imaged to quantify autoregulatory capacity. Figure 1A shows robust dilation and constriction in a representative retinal arteriole from a normotensive rat (first row). In comparison, a representative arteriole from a hypertensive rat (second row) showed less change in diameter. For the same vessel location diameter was quantified and the outcomes are summarised for the group in Fig. 1B as a function of MAP. Under general anaesthesia group average baseline MAP was not significantly different between hypertensive (116.3 ± 12.3 mmHg) and normotensive (125.0 ± 8.3 mmHg) animals (p = 0.5). Figure 1B shows that the change in arteriole diameter during blood pressure manipulation was blunted in chronically hypertensive compared with normotensive rats.

As baseline vessel diameter can vary between eyes, change in width vessel was expressed relative to its own baseline (%) and plotted as a function of change from baseline MAP (∆MAP). In Fig. 1C, the change in vessel width as a function of ΔMAP showed a significant interaction between ΔMAP and chronic BP status (p < 0.001, restricted maximum likelihood analysis, REML), suggesting that the ability of retinal arterioles to buffer the OPP challenge induced by acute BP manipulation was compromised in hypertensive animals. In particular normotensive animals showed significant vasodilation with MAP reductions between −70.0 to −32.5 mmHg. In contrast, hypertensive animals showed no significant vasodilation, with arterioles width remaining stable, regardless of the blood pressure (MAP) level. Only a modest vasoconstriction occurred when MAP increased above baseline levels, which was not significant in either group. It is possible that in some animals BP did not reach sufficiently high levels to induce significant vasoconstriction for our imaging modality to detect. Nevertheless, acute modification of MAP elicited significantly compensatory responses in normotensive but not chronically hypertensive rats.

### Effect of chronic hypertension on vascular morphology

**Figure 2:**
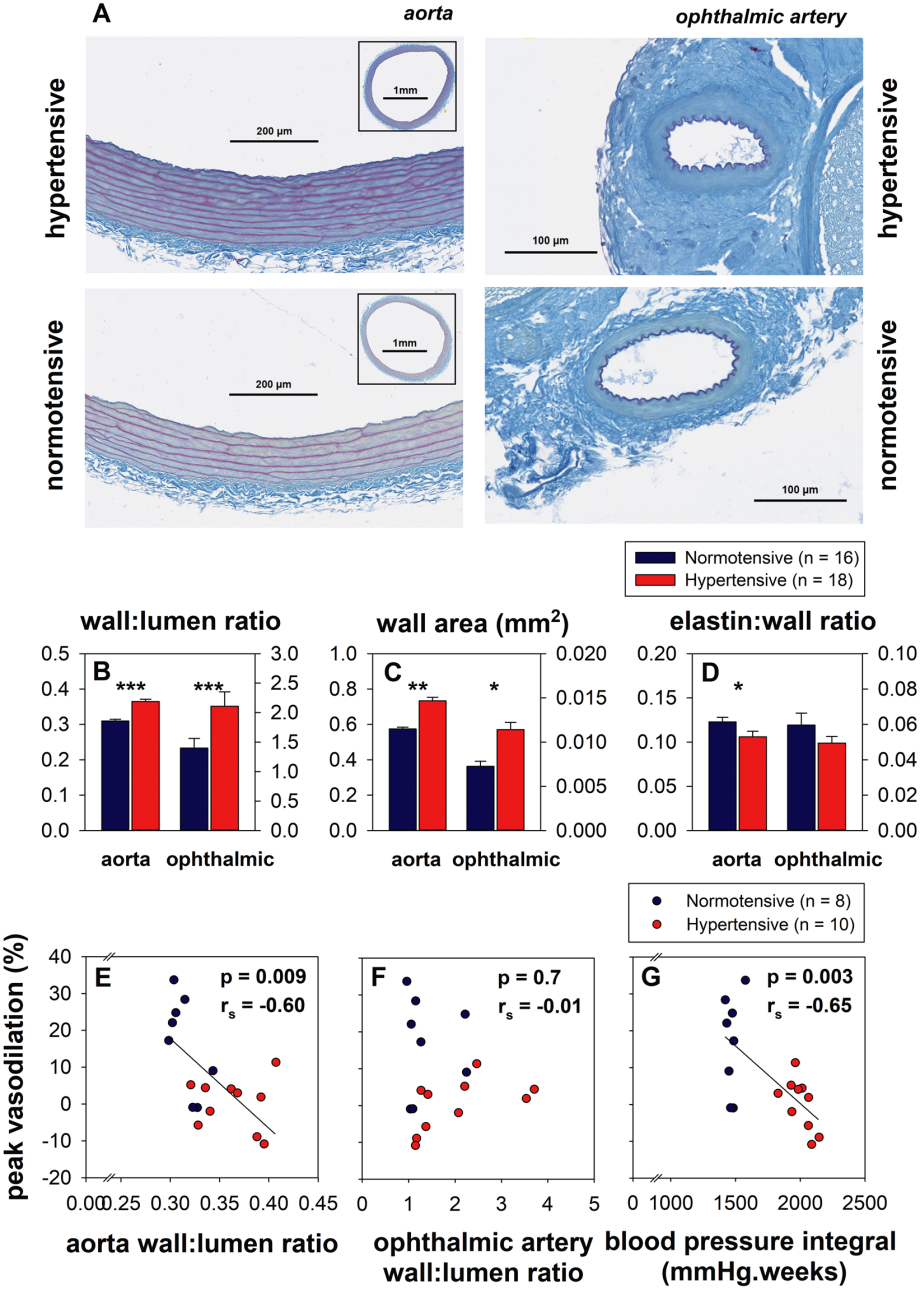
Effect of chronic hypertension on blood vessel morphology. (**A**) Sample hypertensive aorta and ophthalmic arteries from a hypertensive and normotensive rat, with Gomori-Aldehyde Fuchsin staining. (**B**) Aorta and ophthalmic artery wall:lumen ratio. (**C**) Aorta and ophthalmic artery wall area. (**D**) Aorta and ophthalmic artery elastin:wall ratio. (**E**) Correlation between peak vasodilation at low BP and aorta wall:lumen ratio. The solid black line represents the Deming regression (Y = −243.4X + 90.8, r_s_ = −0.6, p = 0.009). (**F**) Correlation between peak vasodilation at low BP and ophthalmic artery wall:lumen ratio. (Deming regression Y = −2.15X + 11.3, r_s_ = −0.01, p = 0.7) (**G**) Correlation between peak vasodilation at low BP and blood pressure integral over 12 weeks. The solid black line represents the Deming regression (Y = −0.031X + 62.7, r_s_ = −0.65, p = 0.003). (*Indicates p < 0.05, **Indicates p < 0.01, ***Indicates p < 0.001).

Aorta and ophthalmic artery tissue was collected from all animals after 12 weeks of systemic hypertension. Figure 2A shows a representative aorta and ophthalmic artery from a hypertensive and normotensive animal. In both the aorta and ophthalmic artery, chronic hypertension resulted in an increase in wall:lumen ratio (WLR, Fig. 2B) and wall area (Fig. 2C). There was a 16% increase in aorta WLR of hypertensive animals from 0.36 ± 0.006 compared to 0.31 ± 0.005 in normotensive animals (p < 0.001). Aortic wall area in treated animals increased by 28%, from 0.73 ± 0.02 mm^2^ compared to 0.57 ± 0.01 mm^2^ in control animals (p < 0.001). Consistent with the changes observed in the aorta, 12 weeks of hypertension resulted in a 50% increase in ophthalmic artery WLR compared to control animals (2.10 ± 0.2 vs 1.40 ± 0.2, p = 0.024). Likewise, the ophthalmic artery wall area of treated animals was also increased by 50% (0.011 ± 8 × 10^–4^ mm^2^ vs 0.0073 ± 5 × 10^–4^ mm^2^, p = 0.002). The aorta elastin:wall ratio (Fig. 2D) was reduced by 8% in hypertensive relative to normotensive animals (0.11 ± 0.006 vs 0.12 ± 0.005, p = 0.047). A 16% reduction in ophthalmic artery elastin:wall ratio was observed in hypertensive relative to normotensive animals (0.05 ± 0.004 vs 0.06 ± 0.007) although this difference did not reach statistical significance (p = 0.2).

As vasodilation in normotensive animals peaked at ∆MAP −55.0 mmHg (Fig. 1C), the % change in vessel diameter at this ∆MAP was used for correlations with blood vessel morphology. Deming regressions show that peak vasodilation was correlated with aorta WLR (Fig. 2E, r_s_ = −0.60, p = 0.009) and long term BP integral (Fig. 2G, r_s_ = −0.65, p = 0.003) but not with ophthalmic artery WLR (p = 0.7). In Fig. 2F, the correlation remains significant (p = 0.049, r_s_ = −0.65) when only hypertensive animals are considered.

Interestingly, while we observed a relationship between aortic morphological changes and retinal autoregulation (Fig. 2E), we were not able to document an association between ophthalmic artery changes and retinal autoregulation (Fig. 2F). One might have expected a stronger relationship between impaired retinal vasodilation and wall thickening in the vessels most proximal to the retina. It is possible that our measurement of wall:lumen ratio in the ophthalmic artery was less sensitive than in the aorta, resulting in greater variability. Another possibility is that loss of autoregulatory capacity may be more related to endothelial dysfunction, than vessel wall thickness. A final possibility is that a reduction of perfusion through the proximal input ophthalmic artery, has already resulted in compensatory vasodilation, thus impeding our attempt to drive further vasodilation by acute BP lowering. This can be seen in Fig. 1B where at what would be considered baseline MAPs of approximated 110–120 mmHg raw vessel width is larger in chronically hypertensive animals.

### Effect of chronic hypertension on retinal function

Given the above vascular deficits, we sought to ascertain whether the ANG II model of chronic hypertension altered retinal function. There was no significant effect of hypertension on photoreceptoral (Rm_P3_), bipolar cell (V_max_) or ganglion cell (positive scotopic threshold response, pSTR) response amplitudes. The oscillatory potential (OP) peak amplitude was reduced at week 8 and week 12, relative to week 4 in hypertensive animals, but was not used to assay the effect of chronic hypertension on retinal susceptibility to chronic IOP challenge, as there was no IOP related effect. These data are presented in Supplementary Materials S2 and S3.

### IOP profile

**Figure 3:**
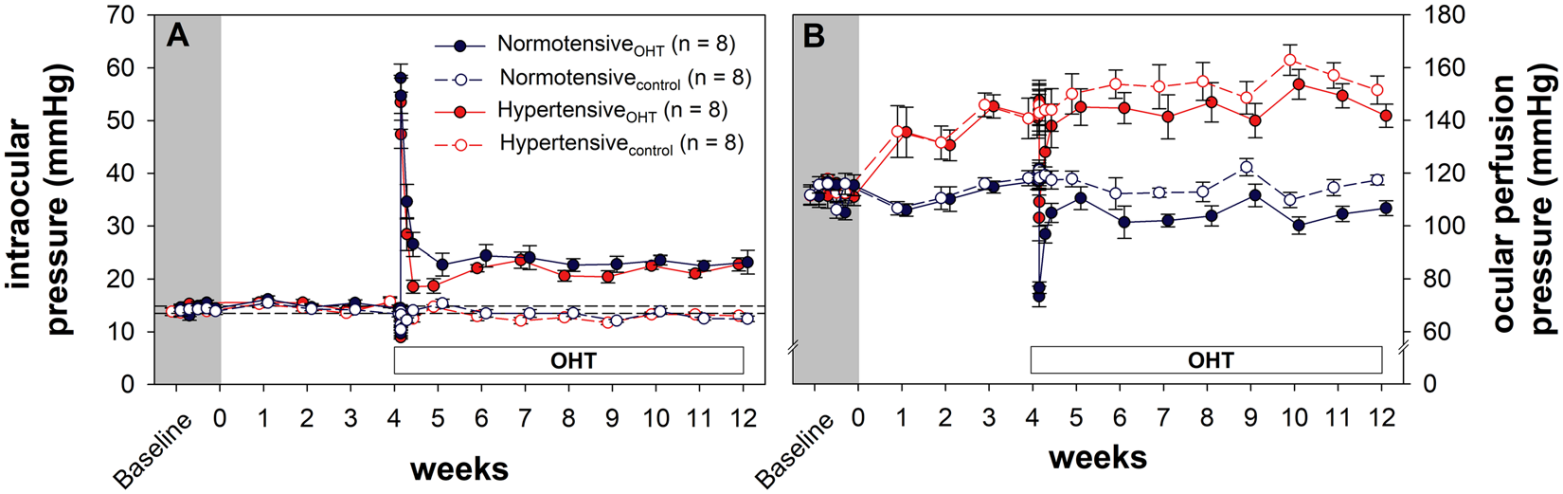
Intraocular pressure profile in animals treated with unilateral circumlimbal suture. (**A**) Effect of circumlimbal suture on IOP (mean ± SEM) in normotensive and hypertensive rats. The broken horizontal reference lines represent the 95% confidence interval of normal IOP derived from baseline measurements across both groups. (**B**) Effect of circumlimbal suture on putative ocular perfusion pressure (mean ± SEM) over 12 weeks in normotensive and hypertensive rats. White bar denotes post circumlimbal suture timepoints. (IOP: intraocular pressure, OHT: ocular hypertension).

The unilateral circumlimbal suture produced a sustained IOP elevation in the treated (ocular hypertension, OHT) eye as shown in Fig. 3A. In normotensive animals, IOP was elevated from a baseline of 14.4 ± 0.5 mmHg to 23.2 ± 1.6 mmHg (week 5–12 average) in OHT eyes (Normotensive_OHT_) (p < 0.001). The fellow eyes (Normotensive_control_) showed no significant change in IOP throughout. Likewise, in the hypertensive animals, IOP was elevated from a baseline of 14.8 ± 0.4 mmHg to 21.5 ± 1.0 mmHg (week 5–12 average) in OHT eyes (Hypertensive_OHT_, p < 0.001), and remained unaltered in control eyes (Hypertensive_control_). In both groups, an initial spike in IOP was observed immediately post suture (normotensive 58.1 ± 2.7 vs hypertensive 53.5 ± 5.1 mmHg, p = 0.4), which recovered to below 40 mmHg within three hours. Given that the different BP groups showed a similar IOP profile, Hypertensive_OHT_ eyes are theoretically afforded a higher OPP than Normotensive_OHT_ eyes (Fig. 3B) based on the calculation of OPP previously described (p < 0.001). One concern about the IOP model is that the initial IOP spike may have reduced OPP to extremely low levels leading to retinal ischemia. The lowest OPP observed during the post-surgical IOP spike was 103.0 ± 8.7 mmHg and 73.4 ± 3.9 mmHg in hypertensive and normotensive animals respectively, which is well above OPP of 30 mmHg where retinal ischemia would occur^16,17^.

### Effect of chronic hypertension on retinal susceptibility to IOP elevation

**Figure 4:**
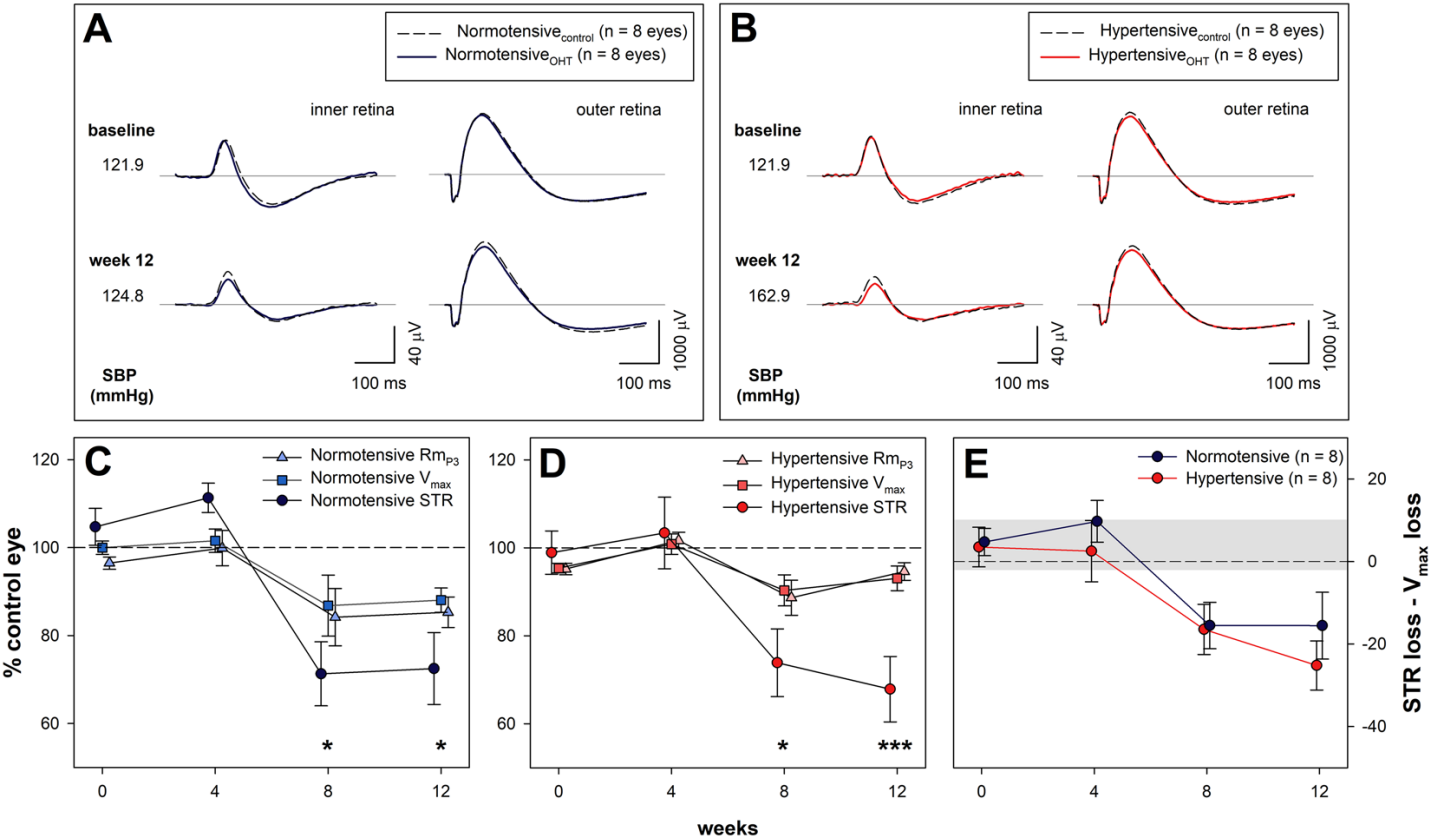
Effect of circumlimbal suture on retinal function in normotensive and hypertensive rats. (**A**,**B**) Group average ERG waveforms at baseline and week 12 in sutured eyes in normotensive (blue trace) and hypertensive (red trace) animals. The dashedblack lines represent the group average waveform in the unsutured control eye in each blood pressure group. Inner retinal responses are shown for a −5.31 logcdsm^−2^ flash. Outer retinal responses are shown for a 2.07 log cdsm^−2^ flash. (**C**) Change in retinal function (mean ± SEM, n = 8) relative to control eyes in normotensive animals. (**D**) Change in retinal function (mean ± SEM, n = 8) relative to control eyes in hypertensive animals. (**E**) Difference between ganglion cell and bipolar cell deficits (pSTR% − V_max_%) in hypertensive and normotensive animals. The shaded region indicates the 95% confidence interval derived from all animals at baseline. *Indicates p < 0.05, ***Indicates p < 0.001 for post-hoc comparisons of change in Rm_P3_ versus STR, and V_max_ versus STR amplitudes at each timepoint.

Figure 4 shows that IOP elevation produced subtle retinal dysfunction which preferentially affected the inner retina. Figure 4A,B shows that electroretinogram (ERG) responses arising from the inner retina were more affected at the 12 week timepoint (i.e. by eight weeks of IOP elevation) than those generated by the outer retina, which are the presynaptic partner neurons to the ganglion cells. This was the case for both normo- and hypertensive groups. Figure 4C,D, expresses this change relative to untreated contralateral control eyes across the 12 weeks of the study, and shows that in normotensive animals IOP elevation significantly reduced photoreceptor (Rm_P3_ −14.7 ± 3.5%), bipolar cell (V_max_ −12.0 ± 2.8%) and ganglion cell (pSTR −27.5 ± 8.2%) amplitudes. Similarly, IOP elevation in hypertensive animals significantly reduced photoreceptor (−5.4 ± 2.0%), bipolar cell (−6.9 ± 2.8%) and ganglion cell (−32.1 ± 7.4%) amplitudes. To consider whether ganglion cell function was more affected than upstream neurons, comparisons were made within each BP group between ERG parameter and time. There was a significant interaction between ERG parameter and time in both normotensive and hypertensive animals (a two-way RM-ANOVA, p < 0.001), with post hoc testing revealing that ganglion cell dysfunction was greater than photoreceptoral and bipolar cell deficits at weeks 8 and 12. Whilst chronic hypertension reduced oscillatory potential amplitudes (Supplementary Fig. S4), chronic IOP elevation did not significantly alter the OPs in both normotensive and hypertensive animals (p = 0.9, data not shown).

The major neuronal classes in the retina, and thus the ERG signal, are arranged in a serial manner, with the through pathway consisting of photoreceptors (Rm_P3_), communicating to bipolar cells (V_max_), which input to ganglion cells (scotopic threshold response, STR). As such, losses in inner retinal function (STR) may reflect damage to retinal ganglion cells and/or reduced input from upstream neurons^33^. One way to account for this potential confound is to express one ERG component relative to its upstream counterpart^34^. In this case we examine the difference between the change ganglion cell output (pSTR %) for a given change in its presynaptic partner neuron the bipolar cells (V_max_ %). Figure 4E shows that that there was no significant difference between BP groups over time (interaction p = 0.8, BP effect, p = 0.3). Despite the marked improvement in OPP in hypertensive animals, there was no functional protection against chronic ocular hypertension. This may suggest that the increase in BP was not sufficient to ameliorate the effects of increased IOP, which is in contrast to previous experimental work, showing that acute BP elevation largely prevented retinal dysfunction caused by IOP elevation^16,27^. A key difference between this study and previous work was the time course of systemic hypertension, with previous studies employing either an acute increase in BP or four weeks of chronic hypertension, whereas the current study considered 12 weeks of chronic hypertension. Perhaps chronic hypertension will become deleterious to retinal function in the context of chronic IOP challenge with even longer durations of blood pressure elevation.

**Figure 5:**
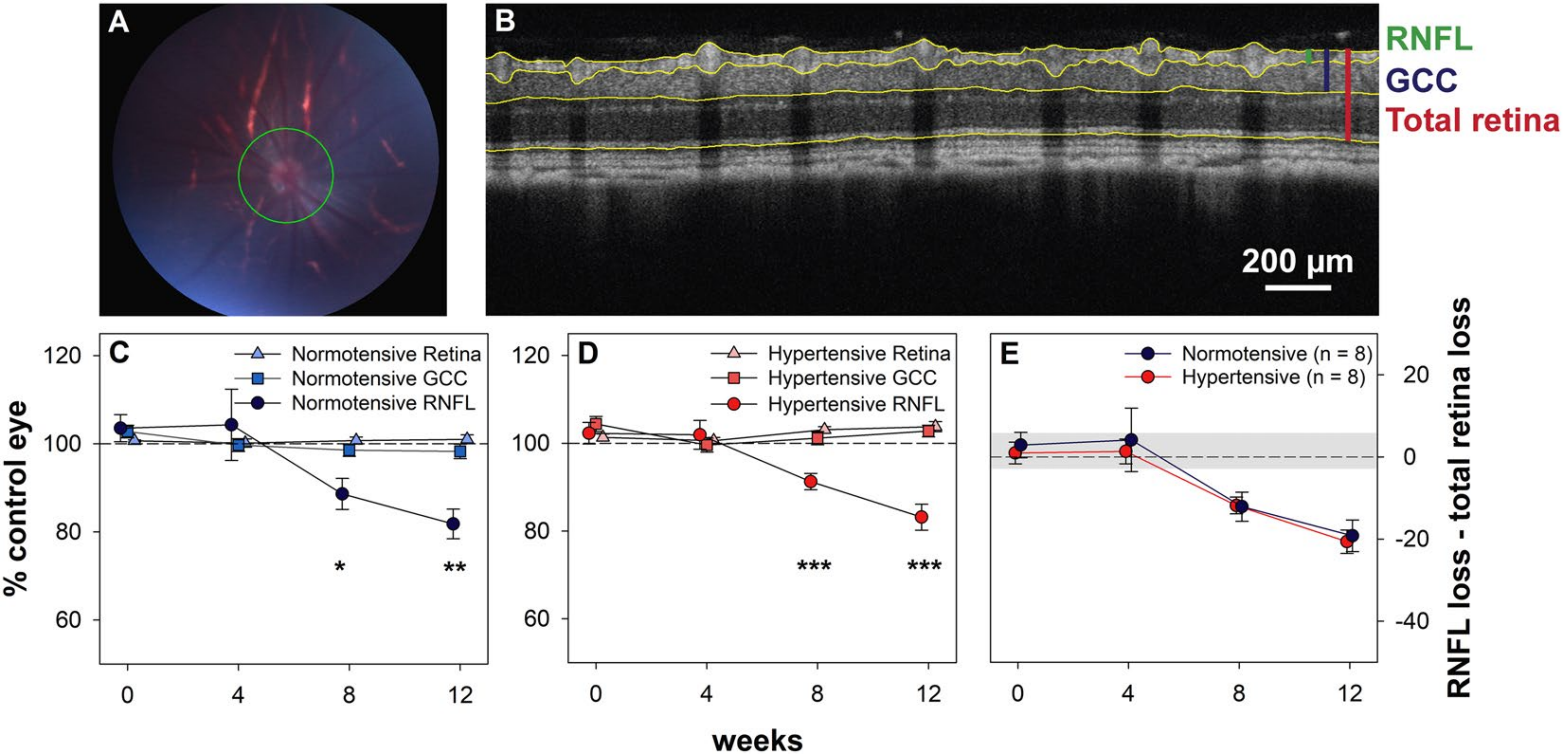
Effect of chronic IOP elevation on retinal layer thickness in normotensive and hypertensive rats. (**A**) Fundus image captured during OCT imaging with green reference ring indicating the position of the OCT scan. (**B**) Linear OCT B-scan (average of 20 B-scans) segmented to demarcate the vitreous interface, border of RNFL and GCL layers, border of IPL and INL layers and the junction between photoreceptor inner and outer segments. Green bar: RNFL; blue bar: GCC; red bar: total retinal thickness. (**C**) Change in retinal layer thickness (mean ± SEM, n = 8) relative to control eyes in normotensive animals. (**D**) Change in retinal layer thickness (mean ± SEM, n = 8) relative to control eyes in hypertensive animals. (**E**) Difference between RNFL change and total retinal thickness change (RNFL% − total retina%) in hypertensive and normotensive animals. The shaded region indicates the 95% confidence interval derived from all animals at baseline. (GCC: ganglion cell complex, RNFL: retinal nerve fibre layer). *Indicates p < 0.05, **Indicates p < 0.01, ***Indicates p < 0.001 for post-hoc comparisons of change in total retinal and RNFL, and GCC and RNFL thickness at each timepoint.

A circular optical coherence tomography (OCT) scan around the ONH (Fig. 5A) was used to assess the effect of chronic ocular hypertension on retinal nerve fibre layer (RNFL), ganglion cell complex (GCC) and total retinal thickness (Fig. 5B). The RNFL represents ganglion cell axons whereas the GCC represent the axonal region as well as the ganglion cell soma and dendritic arbors. Total retinal thickness was not significantly different between OHT treated eyes relative to control eyes at week 12 in either normotensive (+1.0 ± 1.0%) or hypertensive (+3.8 ± 1.2%) animals. When comparing the relative change in total retinal thickness between control and treated eyes in both blood pressure groups, there was no significant interaction (p > 0.9) or blood pressure (p = 0.08) effect, and no significant difference between total retinal thickness relative to control eyes comparing week 0 and week 8 in either hypertensive (p = 0.5) or normotensive (p > 0.9) animals, and comparing week 0 and week 12 in either hypertensive (p = 0.09) or normotensive (p > 0.9) animals. GCC thickness was similar between OHT and control eyes at week 12 (normotensive −1.7 ± 1.6%, hypertensive +2.8 ± 1.3%). There was a marked IOP-induced reduction in RNFL thickness in both normotensive (−18.2 ± 3.4%) and hypertensive animals (−16.8 ± 3.0%) at week 12. Therefore the IOP induced RNFL thinning is progressive across time and selective when compared against total retinal thickness (interaction p < 0.001 in both Fig. 5C and D). These changes were similar between the two BP groups (Fig. 5E, interaction p > 0.9). Similar to the ERG results, in spite of the seemingly better OPP afforded by chronic hypertension, the RNFL was not protected against chronic IOP elevation.

**Figure 6:**
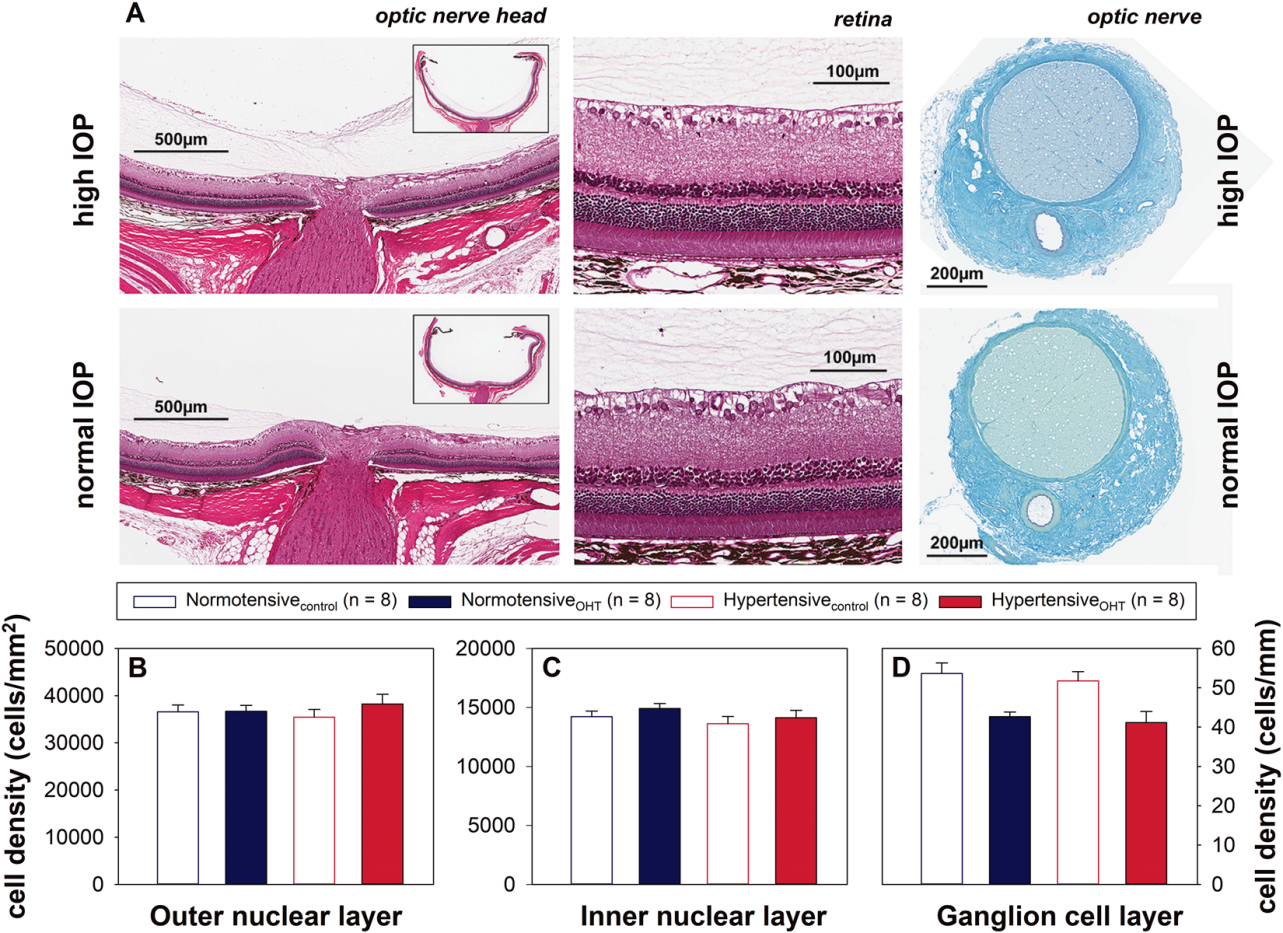
Effect of chronic IOP elevation on retina and optic nerve structure in normotensive and hypertensive rats. (**A**) Representative images from OHT and control eyes of a rat including sagittal section of optic nerve head and posterior eye cup (inset), retina and coronal. (**B**) Cell density (mean ± SEM) in the outer nuclear layer. (**C**) Cell density (mean ± SEM) in the inner nuclear layer. (**D**) Cell density (mean ± SEM) in the ganglion cell layer. (OHT: ocular hypertensive).

The circumlimbal suture model did not produce any gross disturbance of retinal structure or axon bundle arrangement at the ONH (Fig. 6A). Cell density in the outer nuclear layer of OHT eyes was not significantly affected relative to control eyes in either hypertensive (+8.1 ± 3.5%) or normotensive (+0.8 ± 3.5%) animals. Similarly, the cell density of the inner nuclear layer remained unchanged in hypertensive (+4.4 ± 4.5%) and normotensive (5.2 ± 2.6%) animals. Cell density in the ganglion cell layer was significantly reduced however, in both hypertensive (−19.9 ± 5.2%) and normotensive (−18.8 ± 5.5%). Both BP groups were affected similarly, with a two way ANOVA finding no interaction between BP and cell layer (p = 0.4) or BP effect (p = 0.7). A highly significant cell layer effect (p < 0.001) showed that the ganglion cell layer was significantly more affected than outer and inner nuclear layers in both BP groups.

Whilst a significant reduction in cell density of the ganglion cell layer was observed, with histology, no significant reduction in the GCC thickness was observed with OCT. This may be due to the relatively larger contribution of the inner plexiform layer to the overall GCC thickness masking a change in the relatively small contribution of the ganglion cell layer. Alternatively, it is possible that the OCT segmentation did not completely differentiate between the retinal nerve fibre and ganglion cell layers, and thus the reduction in retinal ganglion cell density contributed to the reduction in RNFL thickness observed with OCT.

## Discussion

The salient finding of this study was that despite affording a dramatic increase in putative OPP (Fig. 3B), chronic hypertension did not ameliorate the functional and structural sequelae of chronic IOP elevation (Figs 4E and 5E). The result is in contrast to a previous study in rats that found 4 weeks of hypertension to be protective against acute IOP elevation^27^. One way to explain this would be that that the protective effect of higher blood pressure against IOP elevation is eroded with increasing chronicity of systemic hypertension. Consistent with this idea, He *et al*., did find that 4 weeks of chronic hypertension^27^ was less protective than acute hypertension^16^. Our findings are also broadly consistent with clinical observation. More specifically, the Baltimore Eye Study found systemic hypertension to be protective against glaucoma in younger patients, but increased the risk of glaucoma in older patients, with presumably more advanced effects of long standing blood pressure elevation including arteriosclerosis^22^. Both the Egna-Neumarkt and Los Angeles Latino Eye Studies found both hypo- and hyper-tension to be associated with increased glaucoma risk, suggesting that whilst hypotension can promote poor perfusion of the optic nerve head, hypertension, if long standing, could also lead to compromised optic nerve head perfusion, due to arteriosclerosis and autoregulatory failure^23,25^.

Another key finding from this study was that chronic systemic hypertension significantly reduced retinal autoregulatory capacity during an acute reduction in OPP. Chronic systemic hypertension has been shown to reduce the retinal vascular response to flickering light, a metabolic driver of autoregulation, in humans^31,32^. To our knowledge ours is the first study reporting that chronic hypertension results in impairment of retinal autoregulation driven by systemic blood pressure modification in rats. Impaired pressure induced vasodilation in retinal arterioles might arise from stiffening of the vascular walls as evidence by an increased wall:lumen ratio^35,36^, and vascular endothelial dysfunction via oxidative stress leading to a reduction in nitric oxide bioavailability^37–40^. Although the literature suggests that impaired vasodilation in hypertension is largely due to endothelial dysfunction and consequent reduction in nitric oxide bioavailability^41–43^, as retinal vessels were not examined histologically, structural changes to inner retinal vessel walls could not be ruled out.

The precise mechanism underlying our finding that chronic hypertension did not protect the retina against chronic IOP elevation is not clear. Here we propose two potential mechanisms. Firstly, our observation of impaired vasodilation during acute OPP lowering (Fig. 1) points towards a deficit in retinal autoregulatory capacity. The inner retinal vasculature in animals with chronic hypertension may inadequately compensate for apparently physiological fluctuations in either IOP or blood pressure, leading to short but repeated episodes of insufficient retinal blood supply. This impairment in autoregulation may partially be driven by morphological changes in inner retinal blood vessels or those that feed them (Fig. 2). Thus, compromised autoregulation could increase retinal susceptibility to IOP elevation in spite of increased OPP, by impacting upon the stability of retinal blood flow.

Secondly, the putative improvement in OPP may actually be an artefact of the manner in which OPP is calculated (OPP = MAP_ophthalmic_ – IOP). The equation presumes that peripherally measured BP (brachial artery in humans or tail artery in rodents) faithfully reflects the pressure in the ophthalmic artery and inner retinal vessels. In Fig. 2, we showed a greater magnitude of arterial remodelling in the ophthalmic artery than in the aorta (a larger artery). If remodelling affects smaller arteries more severely, arterial pressure measured in the brachial (or tail) artery may not be a valid surrogate for MAP_ophthalmic_ in patients (or animals) with chronic systemic hypertension. With increasing divergence in vascular remodelling of large and small vessels, the approximation of OPP would become progressively less accurate. OPP as it is currently estimated may need to be modified by to reflective of the severity and chronicity of systemic hypertension. Additionally, OPP estimated in a clinical setting does not capture diurnal variation in OPP and does not consider the capacity of the eye (i.e. autoregulation) to respond to OPP fluctuations. OPP status, which also includes consideration of autoregulatory capacity, could be a more clinically relevant index than the current concept of OPP. The Thessaloniki Eye Study group have used the terms BP and OPP status, to suggest that a normotensive patient and a patient that is normotensive by virtue of anti-hypertensive therapy cannot be regarded in the same way^44–46^. The idea is that in the patient receiving anti-hypertensive therapy, a residual impairment of autoregulatory capacity may persist in spite of having recovered to an ostensibly normal BP level. The Thessaloniki Eye Study Group found an association between lower BP and enlarged cup:disc ratio (a potential sign of glaucoma) and between lower OPP and glaucoma risk, only in patients who used antihypertensive medications^44,46,47^. Patients who had lower BP but were normotensive did not show the same associations. Given that anti-hypertensive therapy has the potential to reverse vascular remodelling and endothelial dysfunction^48,49^, it is possible that given sufficient time, the detrimental effects of hypertension on the ophthalmic vasculature leading to increased glaucoma risk could be reversed with treatment. Our data would support the concept of OPP status as an index of risk, which would encompass IOP and BP in addition to the severity and chronicity of systemic hypertension and the retinal autoregulatory capacity.

Whilst our study shows that chronic hypertension fails to protect retinal structure and function against a chronic IOP challenge in spite of dramatically increasing the putative OPP, certain limitations exist. Firstly, it must be acknowledged that one possible explanation for the apparent similarity between normotensive and hypertensive animals in our study is that circumlimbal suture induced IOP elevation produced a predominantly mechanical injury. This is unlikely, as it is well established in experimental models that vascular injury can damage ganglion cells^6,7,50^, consistent with clinical observations that poor ocular blood flow negatively impacts glaucoma risk^9,11,18,51^. It is difficult to separate the direct compressive effect of raised IOP on retinal ganglion cells and their axons and the indirect vascular effect of reducing tissue perfusion *in vivo*, and it is likely that these processes occur simultaneously. In our study we would expect the mechanical component of an IOP mediated injury to be similar in both groups, but for the hypertensive animals to be protected from the vascular component of the IOP mediated injury.

Secondly, a key limitation of our autoregulation experiment is the use of arteriole diameter as a surrogate measure of blood flow. Several clinical and experimental studies have previously adopted this approach^31,52,53^, however further studies involving blood flow measurement would be pertinent in studying the effects of chronic systemic hypertension on autoregulation. This is particularly important given that a vascular compromise underlies the theory of how chronic hypertension may increase glaucoma risk. As hypertension is largely a disease that affects larger vessels, our study focussed on the first order retinal arterioles, however, a recent study in the human retina shows the capacity of retinal capillaries to alter their diameter in the absence of any change in the diameter of larger “upstream” vessels^54^, raising the possibility that retinal capillaries may compensate to some degree for impairment in the capacity for larger vessels to alter their diameter. Future studies of retinal autoregulation in hypertension, perhaps using optical coherence tomography angiography, should consider the effects of hypertension on the entire trilaminar retinal network.

In summary, we find that despite higher “ocular perfusion pressure” chronic systemic hypertension was not protective for retinal ganglion cells against sustained IOP elevation. This was associated with narrower ophthalmic artery and an impaired capacity for retinal arterial autoregulation. These findings may help to explain why the population based literature that examines glaucoma risk in hypertension remains largely inconclusive. There may also be important implications for clinicians in managing patients with hypertension, particularly when initiating therapy. Our data suggest that retinal autoregulatory capacity is compromised by hypertension, and should BP be lowered too rapidly, patients would be subjected to an OPP challenge in the period where the BP is reduced but residual deficits in autoregulatory capacity persist.

## Materials and Methods

### Animals

All procedures were conducted in accordance with the National Health and Medical Research Council Australian Code of Practice for the care and use of animals for scientific purposes. Ethics approval was obtained from the Howard Florey Institute Animal Experimentation Ethics Committee (Approval number 13-044-UM). In this study male Long Evans rats were used (24–29 weeks old, 300–400 g at the commencement of the study). Animals were housed in a 20 °C facility with a 12 hour light/dark cycle (on at 8 am, 50 lux during light phase), with free access to water and rat chow (Barastoc, Ridley Corporation, Melbourne, VIC, Australia).

A total of 34 rats were used in this 12 week study (normotensive n = 16, hypertensive n = 18). Group-1 (normotensive n = 8, hypertensive n = 10) underwent acute pharmacological BP manipulation and *in vivo* retinal vessel imaging, to assess retinal autoregulatory capacity, after 12 weeks of systemic hypertension. Group-2 (normotensive n = 8, hypertensive n = 8) underwent an IOP elevation surgery on one eye after four weeks, and had retinal function (electroretinography) and *in vivo* retinal structure (optical coherence tomography) measured at four weekly intervals for the duration of the 12 weeks. At the conclusion of 12 weeks of systemic hypertension, all 34 animals underwent cardiac perfusion prior to tissue collection. Samples of the aorta and ophthalmic artery were obtained from all animals. Retina and optic nerve samples were also obtained from animals in Group-2. The experimental timeline is schematised in Supplementary Material S4.

### Blood pressure manipulation and monitoring

Two modalities of BP manipulation and monitoring were used. For chronic BP elevation, all animals were implanted at baseline with an osmotic minipump (2ML4 Alzet Osmotic Pump, Alzet, Cupertino, CA, USA) under the skin to deliver a continuous infusion of angiotensin II (ANG II, hypertensive animals) or normal saline (normotensive controls). The procedure for osmotic minipump implantation is further detailed in Supplementary Material S5. Animals in the hypertensive group received a constant infusion of 150 ng kg^−1^ min^−1^ of ANG II (Auspep, Tullamarine, VIC, Australia). Subcutaneous ANG II infusion is a commonly used drug-induced model of systemic hypertension^35,36^.

All animals underwent weekly conscious systolic BP (SBP) measurements using a tail cuff sphygmomanometer (IN125/R, ADInstruments Pty Ltd, Bella Vista, NSW, Australia). Animals were gently restrained in a custom built restrainer and BP was measured every two minutes over a 30 minute period to derive an average reading. Prior to any BP manipulation surgery, five days of baseline measurements were taken. To minimise the effects of stress in response to restraint, animals were acclimatised to five days of sham BP measurements prior to baseline data collection. SBP was used to estimate mean arterial pressure (MAP), in order to calculate OPP (OPP = MAP – IOP) over the 12 weeks in animals in Group-2. Previous studies have validated the use of tail cuff sphygmomanometry to estimate MAP^55^. Pilot data from our laboratory in Long Evans rats estimated femoral MAPas 0.91 * tail cuff SBP + 16.3.

After 12 weeks of chronic hypertension animals in Group-1 underwent acute pharmacological BP manipulation to challenge autoregulation. Intravenous infusion of sodium nitroprusside 0.6 mg mL^−1^ at a rate of 0.003–0.008 ml min^−1^ was used to reduce MAP to approximately 50 mmHg. A concentration of 0.065 mg mL^−1^ ANG II was infused at a rate of 0.004–0.01 ml min^−1^ to increase MAP to approximately 190 mmHg. The two agents were infused through separate intravenous cannulas. During this procedure, BP was continuously monitored with a cannula in the femoral artery. The surgical procedure for cannulation of the femoral artery and veins is described in Supplementary Material S6.

#### *In vivo* retinal vessel imaging

Retinal vessel diameter change in response to changes in systemic BP is a commonly used surrogate measure of autoregulatory capacity^31,52,53^. Animals in Group-2 underwent retinal vessel imaging during acute BP manipulation. The procedure for retinal vessel imaging and analysis is described in Supplementary Material S7.

#### Intraocular pressure manipulation and monitoring

Animals in Group-2 underwent surgery to chronically elevate IOP after four weeks of systemic hypertension. A circumlimbal suture model of chronic ocular hypertension (OHT), previously described was used^56,57^. This procedure is detailed in Supplementary Material S8.

Rebound tonometry (Tonolab, iCare, Helsinki, Finland) was used for monitoring IOP. Five days of baseline measurements were taken prior to the commencement of the study. Thereafter IOP was measured weekly in all animals in Group-2 for the 12 weeks of the study. At the time of the circumlimbal suture surgery, IOP was measured immediately prior to induction of general anaesthesia, after induction of anaesthesia, and at two minutes, 24 hours and 48 hours post tightening of the circumlimbal suture. Excluding the time points zero and two minutes, all IOP measurements were conducted in awake animals without the use of topical anaesthesia.

#### Electroretinography

A dark-adapted full-field electroretinogram (ERG) was used to assay retinal function in animals in Group-2 at baseline and every 4 weeks for the duration of the study. The ERG is a well established technique for non-invasive objective assessment of retinal function^58,59^. The procedures for signal acquisition and analysis are detailed in Supplementary Material S9. The major components of the ERG waveform reflective of photoreceptoral (Rm_P3_), bipolar cell (V_max_) and retinal ganglion cell (positive scotopic threshold response, pSTR) were assayed in this study. Additionally, oscillatory potential (OP) amplitudes were extracted from the ERG waveform.

#### Optical Coherence Tomography

Spectral domain optical coherence tomography (OCT) (Micron III, Phoenix Research Labs, Pleasanton, CA, USA) was used as a non-invasive assay of retinal thickness. It was performed on animals in Group-2 immediately after each ERG recording. Total retinal thickness, in addition to ganglion cell complex (GCC) and retinal nerve fibre layer (RNFL) thickness was assessed. Acquisition and analysis of OCT signals is described in Supplementary Material S10.

#### Histology

Histology was used to assay the effect of hypertension on arterial morphology and the effect of raised IOP on retinal cell density. At the end of the 12 week study period, all animals underwent cardiac perfusion and tissue harvest. Sections of the aorta and ophthalmic artery were obtained from animals in both groups and stained with Gomori’s Aldehyde Fuchsin staining. Cross sections of retinal tissue were obtained from animals in Group-2 only and stained with Haemotoxylin and eosin. Two 500 μm long sections (one from either side of the optic nerve head) were analysed for each eye. The sections were 5 μm in thickness. Tissue collection, preparation and staining procedures are described further in Supplementary Material S11.

#### Statistical analysis

Group data in this study are presented as the mean ± standard error of the mean (SEM). Data normality (Kolmogorov-Smirnov test) and homogeneity of variances (Bartlett’s test) were established using Prism 6 software (GraphPad Software, La Jolla, CA, USA). A restricted maximum likelihood (REML) analysis^60^, with Bonferroni post hoc testing was used to assess the effect of chronic hypertension on retinal autoregulatory capacity (GenStat software, version 15.2, VSN International, Hemel Hempstead, UK). To analyse the relationships between parameters a Deming regression was used, along with a non-parametric Spearman correlation coefficient (Prism 6). Two way repeated measures ANOVA with Bonferroni post hoc testing was used to compare the effects of chronic hypertension on retinal function, as well as retinal functional and structural susceptibility to chronic IOP elevation (Prism 6).

## Electronic supplementary material

Supplementary material

## References

[1] Quigley, H. A. & Broman, A. T. The number of people with glaucoma worldwide in 2010 and 2020. *Br J Ophthalmol* **90**, 262–267, 90/3/262 10.1136/bjo.2005.081224 (2006). 10.1136/bjo.2005.081224 PMC1856963 16488940

[2] Sommer A (1991). Relationship between intraocular pressure and primary open angle glaucoma among white and black Americans. The Baltimore Eye Survey. Archives of ophthalmology.

[3] Leske MC, Connell AM, Wu SY, Hyman LG, Schachat AP (1995). Risk factors for open-angle glaucoma. The Barbados Eye Study. Archives of ophthalmology.

[4] Quigley HA (2001). The prevalence of glaucoma in a population-based study of Hispanic subjects: Proyecto VER. Archives of ophthalmology.

[5] Leske MC (2007). Predictors of long-term progression in the early manifest glaucoma trial. Ophthalmology.

[6] Orgul S, Cioffi GA, Wilson DJ, Bacon DR, Van Buskirk EM (1996). An endothelin-1 induced model of optic nerve ischemia in the rabbit. Invest Ophthalmol Vis Sci.

[7] Cioffi GA (2004). Chronic ischemia induces regional axonal damage in experimental primate optic neuropathy. Archives of ophthalmology.

[8] Grunwald JE, Piltz J, Hariprasad SM, DuPont J (1998). Optic nerve and choroidal circulation in glaucoma. Invest Ophthalmol Vis Sci.

[9] Plange N, Kaup M, Huber K, Remky A, Arend O (2006). Fluorescein filling defects of the optic nerve head in normal tension glaucoma, primary open-angle glaucoma, ocular hypertension and healthy controls. Ophthalmic & physiological optics: the journal of the British College of Ophthalmic Opticians.

[10] Arend O, Plange N, Sponsel WE, Remky A (2004). Pathogenetic aspects of the glaucomatous optic neuropathy: fluorescein angiographic findings in patients with primary open angle glaucoma. Brain research bulletin.

[11] Jia Y (2014). Optical coherence tomography angiography of optic disc perfusion in glaucoma. Ophthalmology.

[12] He Z, Vingrys AJ, Armitage JA, Bui BV (2011). The role of blood pressure in glaucoma. Clin Exp Optom.

[13] Riva CE, Grunwald JE, Petrig BL (1986). Autoregulation of human retinal blood flow. An investigation with laser Doppler velocimetry. Invest Ophthalmol Vis Sci.

[14] Grunwald JE, Riva CE, Kozart DM (1988). Retinal circulation during a spontaneous rise of intraocular pressure. Br J Ophthalmol.

[15] Riva CE, Hero M, Titze P, Petrig B (1997). Autoregulation of human optic nerve head blood flow in response to acute changes in ocular perfusion pressure. Graefes Arch Clin Exp Ophthalmol.

[16] He Z, Nguyen CT, Armitage JA, Vingrys AJ, Bui BV (2012). Blood pressure modifies retinal susceptibility to intraocular pressure elevation. PLoS One.

[17] Grehn F, Prost M (1983). Function of retinal nerve fibers depends on perfusion pressure: neurophysiologic investigations during acute intraocular pressure elevation. Invest Ophthalmol Vis Sci.

[18] Flammer J (2002). The impact of ocular blood flow in glaucoma. Progress in retinal and eye research.

[19] Khawaja AP, Crabb DP, Jansonius NM (2013). The role of ocular perfusion pressure in glaucoma cannot be studied with multivariable regression analysis applied to surrogates. Invest Ophthalmol Vis Sci.

[20] Hayreh SS, Zimmerman MB, Podhajsky P, Alward WL (1994). Nocturnal arterial hypotension and its role in optic nerve head and ocular ischemic disorders. Am J Ophthalmol.

[21] Leske MC, Wu SY, Hennis A, Honkanen R, Nemesure B (2008). Risk factors for incident open-angle glaucoma: the Barbados Eye Studies. Ophthalmology.

[22] Tielsch JM, Katz J, Sommer A, Quigley HA, Javitt JC (1995). Hypertension, perfusion pressure, and primary open-angle glaucoma. A population-based assessment. Archives of ophthalmology.

[23] Bonomi L (2000). Vascular risk factors for primary open angle glaucoma: the Egna-Neumarkt Study. Ophthalmology.

[24] Dielemans I (1995). Primary open-angle glaucoma, intraocular pressure, and systemic blood pressure in the general elderly population. The Rotterdam Study. Ophthalmology.

[25] Memarzadeh F, Ying-Lai M, Chung J, Azen SP, Varma R (2010). Blood pressure, perfusion pressure, and open-angle glaucoma: the Los Angeles Latino Eye Study. Invest Ophthalmol Vis Sci.

[26] Riva CE, Sinclair SH, Grunwald JE (1981). Autoregulation of retinal circulation in response to decrease of perfusion pressure. Invest Ophthalmol Vis Sci.

[27] He Z, Vingrys AJ, Armitage JA, Nguyen CT, Bui BV (2014). Chronic hypertension increases susceptibility to acute IOP challenge in rats. Invest Ophthalmol Vis Sci.

[28] Grieshaber MC, Mozaffarieh M, Flammer J (2007). What is the link between vascular dysregulation and glaucoma?. Survey of ophthalmology.

[29] Venkataraman ST, Flanagan JG, Hudson C (2010). Vascular reactivity of optic nerve head and retinal blood vessels in glaucoma–a review. Microcirculation.

[30] Strandgaard S (1976). Autoregulation of cerebral blood flow in hypertensive patients. The modifying influence of prolonged antihypertensive treatment on the tolerance to acute, drug-induced hypotension. Circulation.

[31] Nagel E, Vilser W, Lanzl I (2004). Age, blood pressure, and vessel diameter as factors influencing the arterial retinal flicker response. Invest Ophthalmol Vis Sci.

[32] Delles C (2004). Impaired endothelial function of the retinal vasculature in hypertensive patients. Stroke; a journal of cerebral circulation.

[33] Nguyen CT, Vingrys AJ, Wong VH, Bui BV (2013). Identifying cell class specific losses from serially generated electroretinogram components. BioMed research international.

[34] Perlman I (1983). Relationship between the amplitudes of the b wave and the a wave as a useful index for evaluating the electroretinogram. Br J Ophthalmol.

[35] Simon G, Cserep G, Limas C (1995). Development of structural vascular changes with subpressor angiotensin II administration in rats. American journal of hypertension.

[36] Simon G, Illyes G, Csiky B (1998). Structural vascular changes in hypertension: role of angiotensin II, dietary sodium supplementation, blood pressure, and time. Hypertension.

[37] Touyz RM, Schiffrin EL (2008). Reactive oxygen species and hypertension: a complex association. Antioxidants & redox signaling.

[38] Silva BR, Pernomian L, Bendhack LM (2012). Contribution of oxidative stress to endothelial dysfunction in hypertension. Front Physiol.

[39] Koss MC (1999). Functional role of nitric oxide in regulation of ocular blood flow. European journal of pharmacology.

[40] Endemann DH, Schiffrin EL (2004). Endothelial dysfunction. Journal of the American Society of Nephrology: JASN.

[41] Panza JA, Quyyumi AA, Brush JE, Epstein SE (1990). Abnormal endothelium-dependent vascular relaxation in patients with essential hypertension. The New England journal of medicine.

[42] Panza JA, Casino PR, Kilcoyne CM, Quyyumi AA (1993). Role of endothelium-derived nitric oxide in the abnormal endothelium-dependent vascular relaxation of patients with essential hypertension. Circulation.

[43] Bennett MA, Hillier C, Thurston H (1996). Endothelium-dependent relaxation in resistance arteries from spontaneously hypertensive rats: effect of long-term treatment with perindopril, quinapril, hydralazine or amlodipine. Journal of hypertension.

[44] Topouzis F (2006). Association of blood pressure status with the optic disk structure in non-glaucoma subjects: the Thessaloniki eye study. Am J Ophthalmol.

[45] Topouzis F (2013). Association of open-angle glaucoma with perfusion pressure status in the Thessaloniki Eye Study. Am J Ophthalmol.

[46] Harris A (2012). Association of the Optic Disc Structure With the Use of Antihypertensive Medications: The Thessaloniki Eye Study. J Glaucoma.

[47] Topouzis F (2011). Risk factors for primary open-angle glaucoma and pseudoexfoliative glaucoma in the Thessaloniki eye study. Am J Ophthalmol.

[48] Taddei S, Virdis A, Ghiadoni L, Sudano I, Salvetti A (2000). Antihypertensive drugs and reversing of endothelial dysfunction in hypertension. Current hypertension reports.

[49] Schiffrin EL (2012). Vascular remodeling in hypertension: mechanisms and treatment. Hypertension.

[50] Hiraoka M, Inoue K, Ninomiya T, Takada M (2012). Ischaemia in the Zinn-Haller circle and glaucomatous optic neuropathy in macaque monkeys. Br J Ophthalmol.

[51] Yamazaki Y, Drance SM (1997). The relationship between progression of visual field defects and retrobulbar circulation in patients with glaucoma. Am J Ophthalmol.

[52] Tani T, Nagaoka T, Nakabayashi S, Yoshioka T, Yoshida A (2014). Autoregulation of retinal blood flow in response to decreased ocular perfusion pressure in cats: comparison of the effects of increased intraocular pressure and systemic hypotension. Invest Ophthalmol Vis Sci.

[53] Lim M (2013). Systemic associations of dynamic retinal vessel analysis: a review of current literature. Microcirculation.

[54] Duan A, Bedggood PA, Bui BV, Metha AB (2016). Evidence of Flicker-Induced Functional Hyperaemia in the Smallest Vessels of the Human Retinal Blood Supply. PLoS One.

[55] Bunag RD (1973). Validation in awake rats of a tail-cuff method for measuring systolic pressure. J Appl Physiol.

[56] Liu, H. H. *et al*. Chronic ocular hypertension induced by circumlimbal suture in rats. *Invest Ophthalmol Vis Sci*, 10.1167/iovs.14-16009 (2015). 10.1167/iovs.14-16009 25829414

[57] Zhao D (2017). Characterization of the Circumlimbal Suture Model of Chronic IOP Elevation in Mice and Assessment of Changes in Gene Expression of Stretch Sensitive Channels. Front Neurosci.

[58] Weymouth AE, Vingrys AJ (2008). Rodent electroretinography: methods for extraction and interpretation of rod and cone responses. Progress in retinal and eye research.

[59] Robson JG, Frishman LJ (1998). Dissecting the dark-adapted electroretinogram. Documenta ophthalmologica. Advances in ophthalmology.

[60] McGilchrist CA (1993). REML estimation for survival models with frailty. Biometrics.

