# Supplementary material for: Systemic hypertension is not protective against chronic intraocular pressure elevation in a rodent model
Source: Sci Rep. 2018 May 8;8:7107. doi: 10.1038/s41598-018-25264-4 (PMC5940769; doi:10.1038/s41598-018-25264-4)
Supplement: Supplementary file 1 — Supplementary material [file 41598_2018_25264_MOESM1_ESM.pdf]

Title: Systemic hypertension is not protective against chronic IOP elevation in a rodent model

Anna K. Van Koeverden[1], Zheng He[1], Christine T.O. Nguyen[1], Algis J. Vingrys[1], Bang V. Bui[1]*

1 Department of Optometry and Vision Sciences, the University of Melbourne, Parkville, 3010, Victoria, Australia

*** Correspondence:**

Associate Professor Bang Viet Bui

Department of Optometry & Vision Sciences,

The University of Melbourne, Parkville 3010

Victoria, Australia

Ph: +61 3 83447006

Email: bvb@unimelb.edu.au

Word count: 4,030 exclusive of title page, abstract, materials and methods, figure legends and references

SUPPLEMENTARY MATERIAL

S1 Blood pressure profile in subcutaneous ANG II infusion model of hypertension

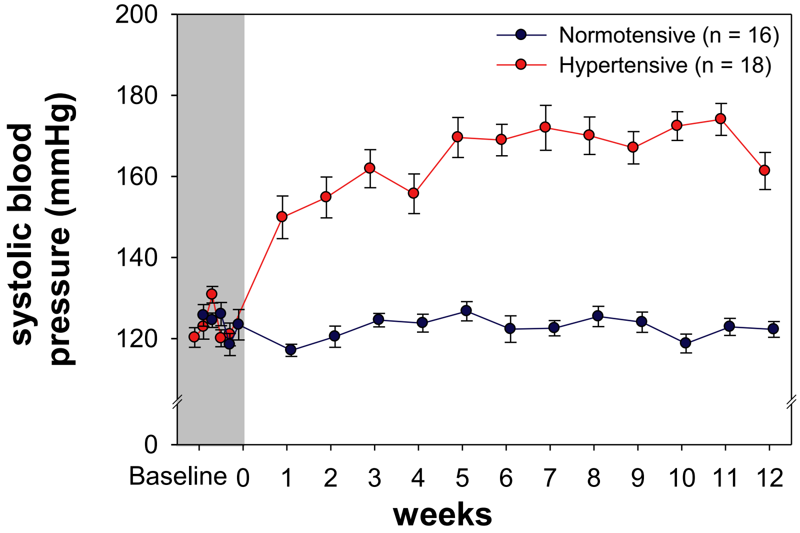

Figure S1: Blood pressure profile. Effect of chronic ANG II infusion on SBP (mean ± SEM) over 12 weeks in normotensive (blue symbols) and hypertensive (red symbols) animals. (SBP: systolic blood pressure, IOP: intraocular pressure).

The average systolic BP (SBP) from weeks 1 – 12 was 164.8 ± 4.6 mmHg and 122.6 ± 2.3 mmHg in hypertensive and normotensive animals, respectively.

A two-way repeated measures ANOVA found no significant interaction between time and group for either hypertensive or normotensive animals (both $p = 0.5$), which we interpret to mean that the different procedures performed in Group-1 and Group-2 did not significantly affect BP.

S2 Effect of 12 weeks of chronic systemic hypertension on retinal function

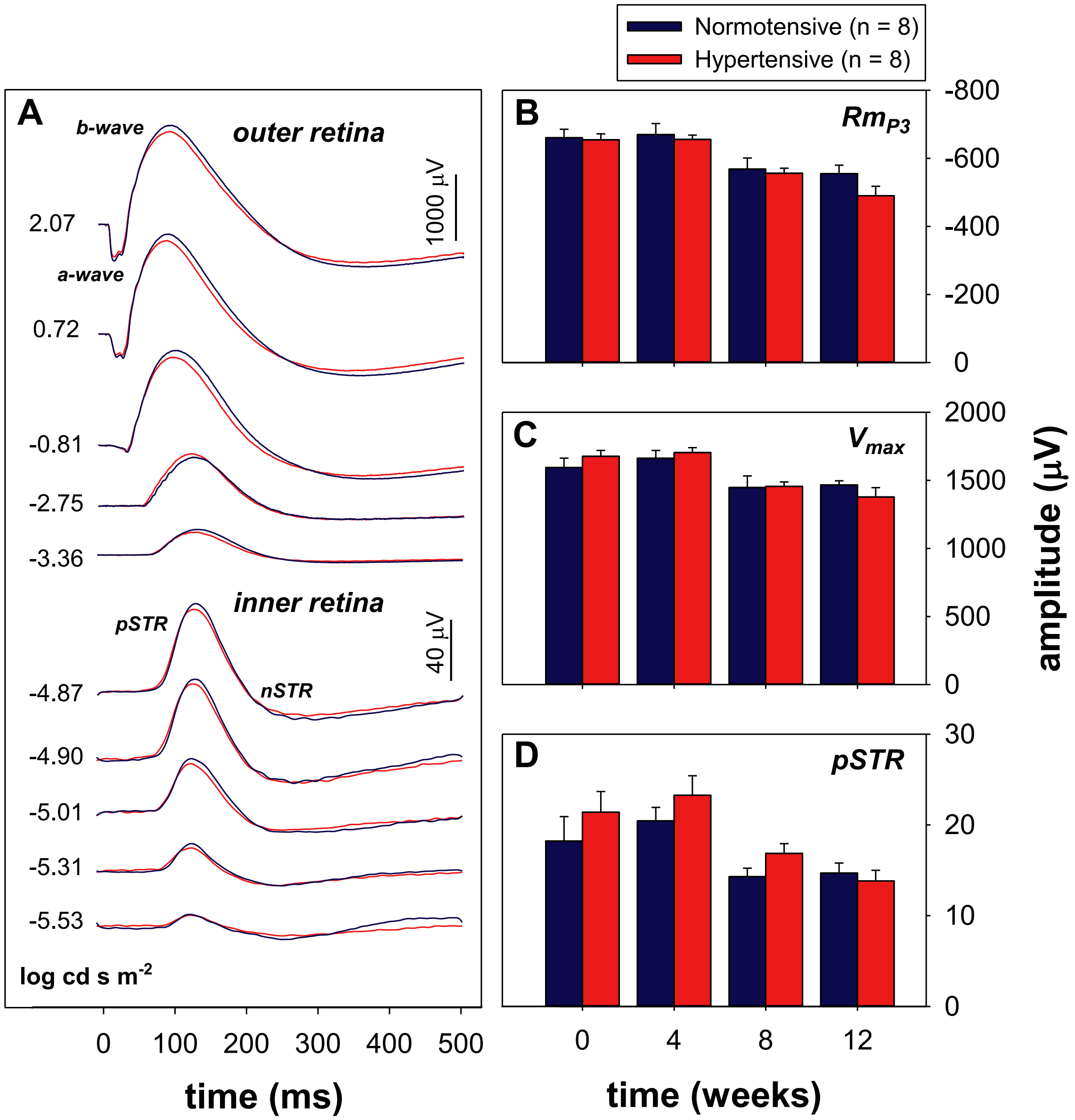

Figure S2: Effect of chronic hypertension on retinal function in ANG II treated (red bars) and saline control (blue bars) animals. **A:** Group average ERG waveforms at week 12. **B:** Photoreceptor amplitude (Rm_{P3}). **C:** Bipolar cell amplitude (V_{max}). **D:** Ganglion cell amplitude (pSTR).

Figure S2A shows group average ERG responses after 12 weeks of ANG II induced hypertension across a range of stimulus energies, demonstrating no gross alteration to retinal function. S2B shows no significant interaction between time and BP ($p = 0.4$), or BP effect ($p =$

0.3) on photoreceptoral amplitude (Rm_{P3}). Similarly, in Figure S2C there was no significant interaction (p = 0.4) or BP (p = 0.2) effect on bipolar cell amplitude (V_{max}). Figure S2D shows that was no significant interaction (p = 0.3) or BP effect (p = 0.4) on ganglion cell function (pSTR). For all three parameters, there was a significant time effect (p<0.001 for all three parameters) which may be due to the effects of ageing and repeated general anaesthesia.

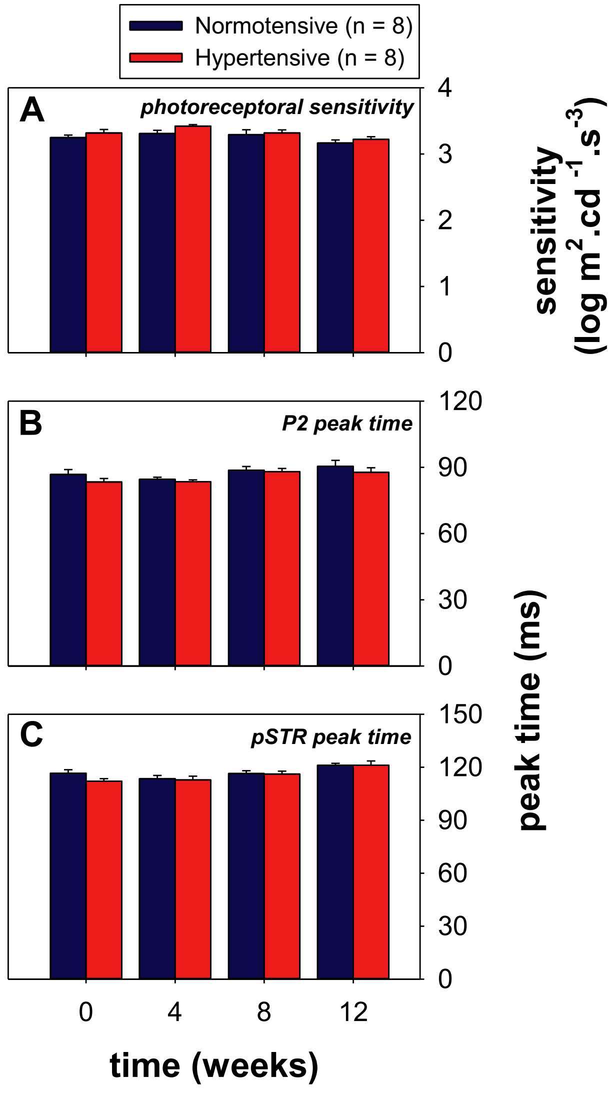

Figure S3: Effect of chronic hypertension on ERG waveform timing in ANG II treated (red bars) and saline control (blue bars) animals. **A:** Photoreceptoral sensitivity (s). **B:** Isolated P2 peak time. **C:** pSTR peak time.

The kinetics of the ERG waveform were also not significantly altered by chronic systemic hypertension (Figure S3). As shown in Figure S3A, there was no significant interaction ($p = 0.4$) or blood pressure ($p = 0.4$) effect on the sensitivity of the of photoreceptoral response, which is reflective of the slope of the fast component of the P3 [1-3]. There was no significant interaction ($p = 0.8$) or blood pressure ($p = 0.6$) effect on the peak time of the isolated P2 after filtering of the oscillatory potentials (Figure S3B). Similarly, for the pSTR response, shown in Figure S3C, there was no significant interaction ($p > 0.9$) or blood pressure effect ($p = 0.1$).

S3 Effect of 12 weeks of chronic systemic hypertension on oscillatory potential (OP) amplitudes

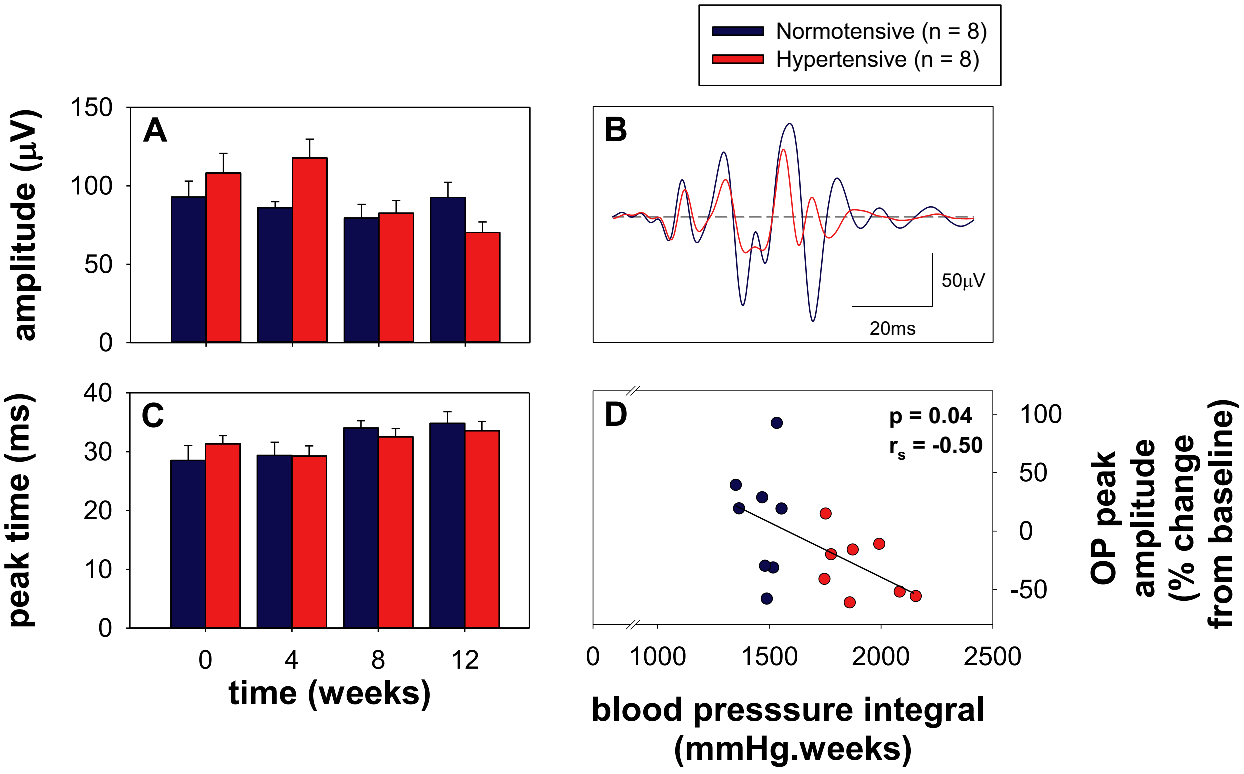

Figure S4: Effect of chronic hypertension on oscillatory potential responses. **A:** Oscillatory potential peak amplitude. **B:** Representative OP waveforms from one hypertensive (red trace) and normotensive (blue trace) animal at week 12. **C:** Oscillatory potential peak time. **D:** Correlation between BP integral over 12 weeks and OP peak amplitude (% change from baseline) found at week 12. The solid black line represents the Deming regression (Y = -0.094X + 148.6, $r_s = -0.50$, p = 0.04).

Figure S4A shows that there was a significant decline in the OP peak amplitude in hypertensive rats (p = 0.02), whereas OP amplitude in normotensive animals did not change appreciably over the 12 weeks. Post hoc analysis revealed that OP amplitude was significantly reduced at weeks 8 (p = 0.02) and 12 (p = 0.003) relative to week 4 in hypertensive animals. Whilst the peak amplitude of the oscillatory potentials was affected by chronic hypertension, there was no significant interaction (p = 0.8) or blood pressure (p = 0.2) effect on the oscillatory potential

peak time (Figure S4C). Representative waveforms collected at week 12 are shown in Figure S4B. Relative change in OP amplitude was significantly correlated with the BP integral across the 12 weeks ($p = 0.04$, Figure S4D).

Oscillatory potentials are considered to be reflective of the integrity of inner retinal circulation [4-7] and have been shown to be altered in human studies in hypertension also[8-10]. Our findings in the rat are consistent with these human studies.

S4 Schematised experimental timeline

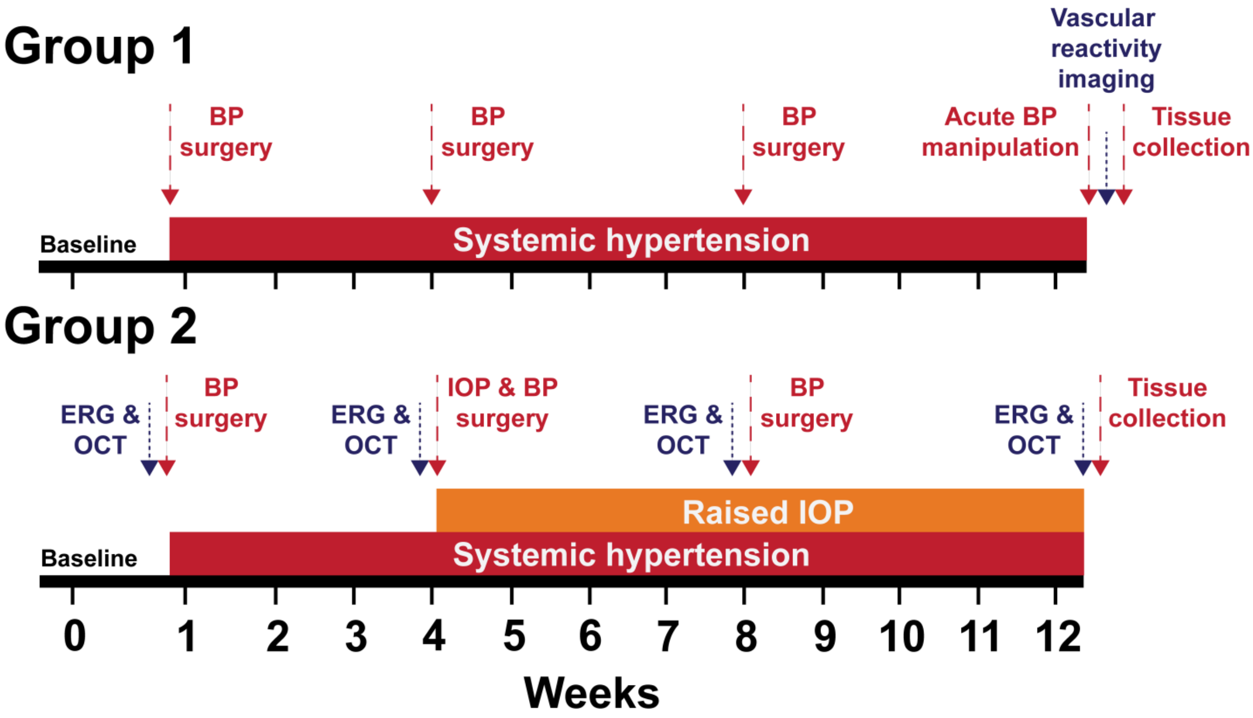

Figure S5: Experimental timeline. In both groups, BP was measured daily for 5 days to establish baseline. After BP elevation, measurements were taken weekly. Dashed red arrows indicate osmotic minipump implantation for BP elevation at weeks 0, 4 and 8. Animals in Group-1 underwent acute blood pressure manipulation at week 12 to assess autoregulatory capacity (blue dashed arrow). All animals underwent cardiac perfusion at the end of experiments prior to tissue collection. Animals in Group-2 underwent circumlimbal suture implantation to elevate IOP at week 4. The circumlimbal suture was left in place for the next 8 weeks. Electroretinography and optical coherence tomography imaging was performed every four weeks in Group-2 (blue dashed arrows). (BP: blood pressure, IOP: intraocular pressure, ERG: electroretinogram, OCT: optical coherence tomography).

S5 Procedure for Alzet Osmotic Pump implantation

Under isoflurane anaesthesia (3% induction, 1.5% maintenance, 1.5 L min^{-1}) and aseptic conditions, a 1 cm incision was made in the skin between the shoulder blades, and a subcutaneous pocket created by blunt dissection. The pump was inserted into the pocket, and the skin closed using interrupted sutures. The 2ML4 pump reservoir holds 2 mL of preloaded

solution and takes four weeks to release its contents. Therefore, a new pump was implanted and the old pump explanted at weeks four and eight.

S6 Procedure for acute pharmacological blood pressure manipulation and simultaneous blood pressure monitoring

Under ketamine:xylazine anaesthesia (60:5 mg kg^{-1}, Troy Laboratory Pty Ltd, Smithfield, NSW, Australia), the femoral artery of one leg, and the femoral vein of two legs were cannulated. A 2 cm incision and blunt dissection of connective tissue were used to expose the femoral artery and vein. A small incision was made into the femoral artery, and a cannula (0.28 and 0.61 mm, inner and outer diameter respectively) filled with heparinised saline (100 IU mL^{-1}, Fisons Pharmaceuticals Pty Ltd, Castle Hill, NSW, Australia) was inserted into the ascending aorta. The arterial cannula was connected to a pressure transducer (Transpac, Abbot Critical Care Systems Illigo, IRE) for continuous blood pressure (BP) monitoring.

Using this same method, the femoral vein in both hind legs was cannulated to allow pharmacological manipulation of BP using intravenous infusion of sodium nitroprusside and ANG II. A concentration of 0.6 mg mL^{-1} sodium nitroprusside was infused at a rate of 0.003 – 0.008 ml min^{-1} to reduce mean arterial pressure (MAP) to approximately 50 mmHg. A concentration of 0.065 mg mL^{-1} ANG II was infused at a rate of 0.004 – 0.01 ml min^{-1} to increase MAP to approximately 190 mmHg.

S7 Procedure for in vivo retinal vessel imaging and analysis

Under general anaesthesia (ketamine:xylazine) and pupil mydriasis (tropicamide), animals were placed on an optical bench setup. A microscope coverslip was used to neutralise the optical power of the cornea. A pellicle beamsplitter (BP2451, Reflectance 45%, Transmittance 55%, Thorlabs Inc., Newton, NJ, USA) reflected light from a Polychrome V light source (577 nm, bandwidth 10 nm, Till Photonics Inc., FEI, Hillsboro, OR, USA) into the eye, using two 75 mm lenses to focus the light onto the rat retina. Light reflected from the retina was directed by the pellicle beamsplitter to a Neo scientific complementary metal oxide semiconductor camera (Andor Technology, Belfast, UK). At each imaging point a stack of 250 images was captured at a rate of 1500 frames per second using MetaMorph software (Molecular Devices, Sunnyvale, CA, USA). Each stack of 250 images was then registered and averaged using ImageJ software. As the Long Evans rat heart rate is approximately 350 beats per minute [11], the image stack encompasses one full cardiac cycle, so any confounding effects of variation in arteriole diameter within the cardiac cycle are removed. Between 30 to 50 image stacks were captured for each animal, and after registration and averaging of each stack, the average images were then registered once more, to allow arteriole diameter to be measured in the same location throughout the BP manipulation procedure. Three to four first order arterioles approximately one disc diameter superior to the optic nerve head were imaged and measured in each animal. The same area was imaged in each animal to minimise any confounding effect of regional variation in blood flow and vessel diameter throughout the retina.

Arteriole diameter was measured using the Diameter plugin in ImageJ software [12], which measures the width of the vessel by plotting the image intensity profile along a line

perpendicular to the vessel. The distance between the half decay and half rise of the intensity profile is taken as the vessel width. The plugin measures the diameter along the line selected by the masked experimenter by re-sampling five times at parallel one pixel intervals. This process was repeated five times for each image to produce a total of 25 diameter measurements for each vessel at each time point, which were then averaged.

S8 Procedure for chronic intraocular pressure (IOP) elevation

Under isoflurane and topical (Alcaine, 0.5% proxymetacaine, Alcon Laboratories, Frenchs Forest, NSW, Australia) anaesthesia, a circumlimbal suture (8/0, nylon) was tied around one randomly selected eye (OHT eye) of each animal approximately 1.5 mm behind the limbus, to elevate IOP by compression of the eyeball. To maximise consistency in the tightness of the suture, the suture was tied as tight as possible in all animals. The suture was secured to the globe by approximately eight subconjunctival anchor points, where the suture was threaded under the conjunctiva, avoiding occlusion of the episcleral veins. The fellow eye was left untreated and served as a within animal control. This rodent glaucoma model was selected over other available models for two key reasons: 1) it reliably produces chronic OHT over several months without the need for multiple procedures facilitating the overlay of chronic OHT with chronic BP elevation, 2) it does not alter the optics of the rodent eye, allowing for repeated measurements of retinal function (electroretinography) and structure (optical coherence tomography). Whilst a large, albeit brief post-surgical IOP spike occurs with use of this model, shown in previous work to peak at two minutes after surgery [13], recent studies where the circumlimbal suture was released 1-2 days post-surgery show no long term detrimental effects of the brief IOP spike [14,15]

S9 Electroretinogram (ERG) stimulus characteristics and procedure for signal acquisition and analysis

Animals were dark adapted overnight and light exposure during set-up was minimised to achieve optimal conditions for eliciting the ganglion cell specific scotopic threshold response [16]. Animals were anaesthetised using intramuscular ketamine: xylazine (60:5 mg kg^{-1}). Topical anaesthesia of the cornea (0.5% proxymetacaine) and pupil mydriasis (0.5% tropicamide, Alcon Laboratories, Frenchs Forest, NSW, Australia) was also performed.

The ERG stimulus was delivered via a LED Ganzfeld sphere (Photometric Solutions International, Oakleigh, VIC, Australia). Stimulus energies ranging from -6.35 to 2.07 log cd s m^{-2} were used to elicit responses from various retinal cell classes. ERG responses were recorded using custom made chlorided silver electrodes. The active electrode was placed on the corneal apex, and was referenced to a ring electrode (inactive) placed around the equator of the same eye. A stainless steel needle electrode (Grass Telefactor, Warwick, RI, USA) inserted subcutaneously into the tail served as the ground. This montage allowed for simultaneous ERG recording from both eyes.

Signals were acquired using Scope™ software (ADInstruments PTY Ltd, Bella Vista, NSW, Australia) using a sampling rate of 4 kHz, 1000 times amplifier gain and hardware band pass filtering (0.3 – 1000 Hz, -3dB with P511 AC amplifier, Grass Technologies, West Warwick, RI, USA). Signals were digitised and saved for post-hoc analysis (ML785 Powerlab 8SP, ADInstruments Pty Ltd, Bella Vista, NSW, Australia).

Photoreceptoral function was assayed by modelling the leading edge of the scotopic a-wave (first electronegative component of the waveform), over an ensemble of responses to three luminous energies (1.55, 1.89 and 2.07 log cd.s.m^{-2}), using a delayed Gaussian function (P3 model)[1-3]. The saturated amplitude (Rm_{P3}) returned by the model is reflective of the number of photoreceptors [17-21]. The second positive deflection of the waveform is known as the b-wave, is a summation of the negative P3 and the positive P2 components. Superimposed on the rising limb of the b-wave are a number of high frequency, low amplitude oscillations, known as oscillatory potentials (OPs), which originate in the inner plexiform layer[5,22,23]. Subtraction of the P3 model from the ERG waveform reveals the P2-OP complex. A bandpass filter (Butterworth Filter, 55 and 215 Hz, -3 dB) was used to extract the OPs, revealing the isolated P2 amplitude, reflective of ON-bipolar cell activity [24-26]. The relationship between stimulus energy and P2 amplitude was modelled using a saturating hyperbolic function, which returns a maximal response amplitude (V_{max}), reflective of bipolar cell function. A range of 18 stimulus energies between -6.35 and 2.07 log cd.s.m^{-2} were used to model the P2 response to extract V_{max} [27-29]. A twin flash paradigm at 2.07 log cd.s.m^{-2} was employed to extract the isolated rod contribution to the P2 waveform [30-32]. Retinal ganglion cell function was assayed using the positive scotopic threshold response (pSTR), measured using very dim stimulus energies [33-35]. In this study, the amplitude at 110 ms after stimulus onset in response to a -5.31 log cd s m^{-2} flash was used to indicate ganglion cell function.

S10 Procedure for optical coherence tomography (OCT) signal acquisition and analysis

Animals were placed on an alignment stage and Genteal Gel (Novartis, North Ryde, NSW, Australia) was applied to the eye as a coupling interface between the eye and the rat-specific

OCT objective lens. A circular B-scan located 1 mm from the centre of the optic nerve was used, consisting of 1024 A-scans captured at a speed of 20,000 A-scans per second. To improve signal:noise ratio 20 B-scans were acquired which were then registered and averaged using ImageJ software (National Institutes of Health, Bethesda, MD, USA).

OCT images were then manually segmented by a masked experimenter using Image-Pro Plus software (MediaCybernetics, Inc., Rockville, MD, USA) to extract the retinal nerve fibre layer (RNFL), ganglion cell complex (GCC) and total retinal thicknesses. Layer thickness was measured by plotting the layer thickness at each pixel as a frequency histogram using Excel Software (Microsoft, Redmond, WA, USA) and modelling the distribution with a Gaussian function, where the peak position was taken as the layer thickness. In the case of the RNFL, the frequency distribution showed two distinct peaks, with the first and larger peak corresponding to the RNFL thickness, and the second and smaller peak corresponding to the locations of retinal blood vessels. To avoid any potential confounding effect of systemic hypertension mediated change in blood vessel size, RNFL thickness was modeled using two Gaussian functions, where the peak position of the first curve was taken as the average RNFL thickness.

S11 Procedure for tissue collection, preparation and staining

Under deep general anaesthesia (ketamine:xylazine), 60 mL of room temperature 0.1 M phosphate buffered saline was injected into the left ventricle via open thorax to perfuse the animal, followed by 30 mL of 4% Paraformaldehyde (Electron Microscopy Sciences, Hatfield, PA, USA) in 0.1 M phosphate buffered saline. A 2 cm segment of thoracic aorta at the level of the diaphragm was dissected and post fixed in 10% neutral buffered formalin with the distal 0.5

mm used for analysis. Both eyes were eneucleated, leaving 5 – 8 mm of the optic nerve attached to the globe. After enucleation the anterior chamber and the vitreous body were removed. The posterior eye cups were post fixed in Davidson's fixative for 24 hours before being transferred to 10% neutral buffered formalin for embedding and sectioning.

Prior to embedding, tissues were dehydrated in three washes of 70%, 90% and 100% ethanol, then cleared with xylene. Tissues were then embedded in paraffin wax for sectioning. Coronal sections of the retrobulbar optic nerve were obtained at a distance of 2 mm from the sclera, to assay the ophthalmic artery, after which the paraffin block was re-embedded so that the retina could be sectioned the sagittal plane. Sections of retinal tissue were only obtained for animals in Group-1. Sections (5 µm thickness) were mounted and air dried and then deparaffinised and rehydrated prior to staining. The aorta and optic nerve (containing the ophthalmic artery) sections were stained with Gomori's Aldehyde-Fuchsin, with Indigo-Carmine-picric acid counterstaining to increase the visibility of elastin fibres. Haemotoxylin and eosin staining was used for retinal sections.

Slides were scanned at 40 times magnification using an AperioScanScope® CS (Leica Microsystems Pty Ltd, North Ryde, NSW, Australia) and analysed using Image-Pro Plus software. To quantify blood vessel wall:lumen ratio and wall area, the boundaries of the blood vessel lumen and tunica media were defined. Elastin:wall ratio was measured by quantifying the area of Gomori Aldehyde-Fuchsin staining within the wall relative to the wall area. Retinal cell density in the outer nuclear, inner nuclear and ganglion cell layers was quantified along two 500 µm sections on either side of the optic nerve head. The width of the area of interest was 40 and

60 μm for the inner and outer nuclear layers respectively. Cell density in the outer and inner nuclear layers was performed automatically using Image-Pro Plus software (version 6.0, MediaCybernetics, Inc., Rockville, MD, USA). Cell density in these layers is reported in cells mm^{-2}. Cell nuclei in the ganglion cell layer do not stain strongly with haematoxylin, therefore automated cell counting was deemed unreliable, and cell nuclei in this layer were counted manually. As the ganglion cell layer is a monolayer of cells, ganglion cell layer density is reported in cells/length of section (cells mm^{-1}). The retina within 200 μm of the scleral canal opening was excluded from analysis due to the presence of large retinal blood vessels in this region. Cell counting was performed by a single experimenter who was blinded to both BP and IOP treatments.

A limitation of the histological methodology is that it samples only a small portion of the total retinal ganglion cell population of the retina. Cross sectional sampling was chosen as the effect of the circumlimbal suture OHT model on cell density in the outer and inner nuclear layers has not previously been reported. Subsequent studies using this model should employ retinal ganglion cell specific staining in flat mounted retinas to allow for more accurate and regional analysis of retinal ganglion cell density [14].

REFERENCES

1 Lamb, T. D. & Pugh, E. N., Jr. A quantitative account of the activation steps involved in phototransduction in amphibian photoreceptors. *The Journal of physiology* **449**, 719-758 (1992).

2 Hood, D. C. & Birch, D. G. Rod phototransduction in retinitis pigmentosa: estimation and interpretation of parameters derived from the rod a-wave. *Invest Ophthalmol Vis Sci* **35**, 2948-2961 (1994).

3 Hood, D. C. & Birch, D. G. Phototransduction in human cones measured using the alpha-wave of the ERG. *Vision research* **35**, 2801-2810 (1995).

4 Wachtmeister, L. Some aspects of the oscillatory response of the retina. *Progress in brain research* **131**, 465-474 (2001).

5 Wachtmeister, L. Oscillatory potentials in the retina: what do they reveal. *Progress in retinal and eye research* **17**, 485-521 (1998).

6 Luu, C. D., Szental, J. A., Lee, S. Y., Lavanya, R. & Wong, T. Y. Correlation between retinal oscillatory potentials and retinal vascular caliber in type 2 diabetes. *Invest Ophthalmol Vis Sci* **51**, 482-486, doi:10.1167/iovs.09-4069 (2010).

7 Vadala, M., Anastasi, M. & Lodato, G. Transient reduction of the ocular perfusion pressure and the oscillatory potentials of the ERG. *Vision research* **45**, 1341-1348, doi:10.1016/j.visres.2004.10.027 (2005).

8 Ravalico, G. *et al.* Oscillatory potentials of the electroretinogram in hypertensive patients with different antihypertensive treatment. *Documenta ophthalmologica. Advances in ophthalmology* **94**, 321-326 (1997).

9 Ravalico, G. *et al.* Oscillatory potentials in subjects with treated hypertension. *Ophthalmologica. Journal international d'ophtalmologie. International journal of ophthalmology. Zeitschrift fur Augenheilkunde* **209**, 187-189 (1995).

10 Bellini, G. *et al.* Oscillatory potentials of the electroretinogram in hypertensive patients. *Hypertension* **25**, 839-841 (1995).

11 Tomlinson, K. C., Gardiner, S. M. & Bennett, T. Blood pressure in streptozotocin-treated Brattleboro and Long-Evans rats. *The American journal of physiology* **258**, R852-859 (1990).

12 Fischer, M. J., Uchida, S. & Messlinger, K. Measurement of meningeal blood vessel diameter in vivo with a plug-in for ImageJ. *Microvascular research* **80**, 258-266, doi:10.1016/j.mvr.2010.04.004 (2010).

13 Liu, H. H. *et al.* Chronic ocular hypertension induced by circumlimbal suture in rats. *Invest Ophthalmol Vis Sci*, doi:10.1167/iovs.14-16009 (2015).

14 Zhao, D. *et al.* Characterization of the Circumlimbal Suture Model of Chronic IOP Elevation in Mice and Assessment of Changes in Gene Expression of Stretch Sensitive Channels. *Front Neurosci* **11**, 41, doi:10.3389/fnins.2017.00041 (2017).

15 Liu, H. H. & Flanagan, J. G. A Mouse Model of Chronic Ocular Hypertension Induced by Circumlimbal Suture. *Invest Ophthalmol Vis Sci* **58**, 353-361, doi:10.1167/iovs.16-20576 (2017).

16 Naarendorp, F., Sato, Y., Cajdric, A. & Hubbard, N. P. Absolute and relative sensitivity of the scotopic system of rat: electroretinography and behavior. *Visual neuroscience* **18**, 641-656 (2001).
17 Brown, K. T. & Wiesel, T. N. Intraretinal recording with micropipette electrodes in the intact cat eye. *The Journal of physiology* **149**, 537-562 (1959).
18 Brown, K. T. & Wiesel, T. N. Localization of origins of electroretinogram components by intraretinal recording in the intact cat eye. *The Journal of physiology* **158**, 257-280 (1961).
19 Baylor, D. A., Matthews, G. & Nunn, B. J. Location and function of voltage-sensitive conductances in retinal rods of the salamander, Ambystoma tigrinum. *The Journal of physiology* **354**, 203-223 (1984).
20 Bush, R. A. & Sieving, P. A. A proximal retinal component in the primate photopic ERG a-wave. *Invest Ophthalmol Vis Sci* **35**, 635-645 (1994).
21 Green, D. G. & Kapousta-Bruneau, N. V. A dissection of the electroretinogram from the isolated rat retina with microelectrodes and drugs. *Visual neuroscience* **16**, 727-741 (1999).
22 Korol, S., Leuenberger, P. M., Englert, U. & Babel, J. In vivo effects of glycine on retinal ultrastructure and averaged electroretinogram. *Brain research* **97**, 235-251 (1975).
23 Ogden, T. E. The oscillatory waves of the primate electroretinogram. *Vision research* **13**, 1059-1074 (1973).
24 Brown, K. T. The electroretinogram: its components and their origins. *Vision research* **8**, 633-677 (1968).
25 Robson, J. G., Maeda, H., Saszik, S. M. & Frishman, L. J. In vivo studies of signaling in rod pathways of the mouse using the electroretinogram. *Vision research* **44**, 3253-3268, doi:10.1016/j.visres.2004.09.002 (2004).
26 Stockton, R. A. & Slaughter, M. M. B-wave of the electroretinogram. A reflection of ON bipolar cell activity. *J Gen Physiol* **93**, 101-122 (1989).
27 Fulton, A. B. & Rushton, W. A. The human rod ERG: correlation with psychophysical responses in light and dark adaptation. *Vision research* **18**, 793-800 (1978).
28 Baylor, D. A., Nunn, B. J. & Schnapf, J. L. The photocurrent, noise and spectral sensitivity of rods of the monkey Macaca fascicularis. *The Journal of physiology* **357**, 575-607 (1984).
29 Awatramani, G., Wang, J. & Slaughter, M. M. Amacrine and ganglion cell contributions to the electroretinogram in amphibian retina. *Visual neuroscience* **18**, 147-156 (2001).
30 Friedburg, C., Thomas, M. M. & Lamb, T. D. Time course of the flash response of dark- and light-adapted human rod photoreceptors derived from the electroretinogram. *The Journal of physiology* **534**, 217-242 (2001).
31 Nixon, P. J., Bui, B. V., Armitage, J. A. & Vingrys, A. J. The contribution of cone responses to rat electroretinograms. *Clin Experiment Ophthalmol* **29**, 193-196 (2001).
32 Weymouth, A. E. & Vingrys, A. J. Rodent electroretinography: methods for extraction and interpretation of rod and cone responses. *Progress in retinal and eye research* **27**, 1-44, doi:10.1016/j.preteyeres.2007.09.003 (2008).
33 Naarendorp, F. & Sieving, P. A. The scotopic threshold response of the cat ERG is suppressed selectively by GABA and glycine. *Vision research* **31**, 1-15 (1991).
34 Frishman, L. J. *et al.* The scotopic electroretinogram of macaque after retinal ganglion cell loss from experimental glaucoma. *Invest Ophthalmol Vis Sci* **37**, 125-141 (1996).

35 Bui, B. V. & Fortune, B. Ganglion cell contributions to the rat full-field electroretinogram. *The Journal of physiology* **555**, 153-173, doi:10.1113/jphysiol.2003.052738 (2004).
